# Understanding cassava varietal preferences through pairwise ranking of *gari‐eba* and *fufu* prepared by local farmer–processors

**DOI:** 10.1111/ijfs.14862

**Published:** 2020-11-20

**Authors:** Béla Teeken, Afolabi Agbona, Abolore Bello, Olamide Olaosebikan, Emmanuel Alamu, Michael Adesokan, Wasiu Awoyale, Tessy Madu, Benjamin Okoye, Ugo Chijioke, Durodola Owoade, Maria Okoro, Alexandre Bouniol, Dominique Dufour, Clair Hershey, Ismail Rabbi, Busie Maziya‐Dixon, Chiedozie Egesi, Hale Tufan, Peter Kulakow

**Affiliations:** ^1^ International Institute of Tropical Agriculture Ibadan PMB 5320 Nigeria; ^2^ International National Root Crops Research Institute (NRCRI) Umudike, Umuahia PMB 7006 Nigeria; ^3^ Department of Food Science & Technology Kwara State University Malete Kwara State PMB 1530 Nigeria; ^4^ Laboratoire de Sciences des Aliments Faculté des Sciences Agronomiques Université d’Abomey‐Calavi Jéricho 03 BP 2819 Benin; ^5^ CIRAD UMR QUALISUD Cotonou 01 BP 526 Benin; ^6^ Qualisud CIRAD Montpellier SupAgro Université d'Avignon Université de La Réunion Université Montpellier Montpellier, 34398 France; ^7^ CIRAD UMR QUALISUD Montpellier F‐34398 France; ^8^ Private consultant PO Box 4 Flinton PA 16640 USA; ^9^ College of Agriculture and Life Sciences Cornell University 215 Garden Avenue Ithaca NY 14853 USA

**Keywords:** Breeding, cassava, farmer–processors, food quality, fufu, gari, improved varieties, landraces, Nigeria, participatory variety selection methods

## Abstract

Within communities in Osun and Imo States of Nigeria, farmer–processors grew and processed a diverse set of improved and landrace cassava varieties into the locally popular foods, *gari*, *eba* and *fufu*. Local and 15 main varieties were grown in a ‘mother and baby trials’ design in each state. Mother trials with three replications were processed by farmer–processors renown in their community for their processing skills. Baby trials were managed and processed by other farmer–processors. The objective was to identify food quality criteria to inform demand‐led breeding to benefit users, especially women, given their key roles in processing. Farmer–processors evaluated the overall quality of fresh roots and derived food products through pairwise comparisons. Improved varieties had higher fresh and dry root yield. Overall, landraces ranked first for quality of gari and eba, but several improved varieties were also appreciated for good quality. Landraces in Osun had higher gari yield and a higher swelling power compared to improved varieties. Colour (browning), bulk density, swelling power, solubility and water absorption capacity were the criteria most related to food product ranking by farmer–processors. Evaluation of varieties under farmer–processors’ conditions is crucial for providing guidance to breeders on critical selection criteria.

## Graphical abstract

Using a socially inclusive ‘mother baby trial’ approach, farmer processors in two states in Nigeria grew and processed a diverse set of cassava varieties and evaluated the root and food product quality and provided quality criteria through pairwise ranking. This evaluation was linked to fresh roots agronomic data as well as the laboratory measurements on the food product *gari* to inform breeding.

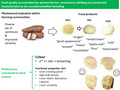

## Introduction

Cassava is a major staple crop in Nigeria, mostly processed into food products by small‐ or medium‐scale farmers and processors (IITA, 2012; Onyenwoke & Simonyan, 2014; Forsythe *et al*., 2016). Cassava farmers often add significant value to the crop by processing the roots. The most common products are *gari*, *eba* and *fufu*. Processing is mainly done by women, while both men and women do cassava farming (Curran *et al*., 2009; Walker *et al*., 2014; Taiwo & Fasoyiro, 2015; Teeken *et al*., 2018). In Africa, industrial uses of the crop, for example starch, ethanol and other applications, are still at relatively low levels but growing (Taiwo, 2006; Echebiri & Edaba, 2008; Osuji *et al*, 2017).

Gari (or cassava semolina) is a gelatinised, fine to coarse granular flour made from grated and fermented cassava roots. Gari is an important marketed product because of its storability and its precooked nature, allowing it to be prepared for consumption in minutes using cold water (as drinkable gari) or hot water to make the dough‐like product called eba in Nigeria. Gari is made by peeling, washing and grating cassava, fermenting (depending on the region), and dewatering to obtain a mash, after which the mash is sieved and toasted to gari. Fufu, also known as *akpu*, is made by retting (soaking) the peeled roots for 3–5 days (until the roots are softened), removing fibres and unsoftened parts of the roots and leaving the resulting mash in permeable sacks or strainers to allow the drainage of excess water after which the fufu is cooked over a fire (Hahn, 1989). Cooked fufu is especially liked as its texture resembles that of pounded yam, the traditional staple food before cassava became important in Nigeria (Korieh, 2010). Most often farmer–processors make both gari and fufu from the same cassava variety. Although our primary focus is on gari and eba, we also evaluated fufu quality for this reason.

Breeding programmes have focused on developing varieties suitable for a range of agroecological zones. Primary criteria for selection have been yield, dry matter content and resistance to common diseases such as cassava brown streak (for Eastern Africa) and cassava mosaic disease. These improvements have driven a moderately successful level of adoption (Alene *et al*., 2015; Walker & Alwang, 2015; Oparinde *et al*., 2016; Wossen *et al*., 2017; Thiele *et al*., 2020). However, although many farmers grow improved varieties, the total area they allocate to them is still limited. Additionally, many of these improved varieties are unreleased and unpromoted clones ‘escaped’ from breeders’ trials and disseminated among farmers (Thiele *et al*., 2020). Varieties are often released without consideration of the standards needed to process quality food products such as gari and fufu. By comparison, farmers and processors typically use landraces that are the result of long trajectories of selection by farmers and processors, which includes quality traits. Recent research has indicated the major importance of food quality traits within the adoption of new varieties (Nduwumuremyi *et al*., 2016; Wossen *et al*., 2017; Teeken *et al*., 2018). Research has also shown the influence of varieties on the gari product quality (Komolafe & Arawande, 2010; Sanoussi *et al*., 2015; Awoyale *et al*., 2020; Akely *et al*., 2020).

This article compares a diverse set of clones, including landraces and improved varieties, to understand criteria for food product quality of smallholder farmer–processors using a participatory variety selection ‘mother–baby’ trial design.

The specific objectives of the study were to:

1. investigate the suitability of landraces and improved varieties for gari and fufu processing as evaluated by smallholder farmer–processors;
2. establish criteria used by farmer–processors to evaluate the food product qualities and relate these to the physico‐chemical and functional properties of gari;
3. investigate the relationship between fresh roots quality characteristics, and physico‐chemical and functional properties of gari.

## Materials and methods

### Participatory site selection

Based on the results of the cassava monitoring survey (CMS) by Wossen *et al*. (2017), two of the highest cassava producing and consuming states of Nigeria, Osun and Imo were selected to represent two different agroecological zones and two different gari producing cultures. In Osun, within the forest/savanna transition agroecology, long fermentation time of gari is practised: four‐day wet fermentation after which the mash is pressed for half a day, resulting in a very sour gari. In Imo, within the humid forest ecology, a shorter fermentation is practised: mash is pressed directly after grating and left under the press for two days. Often palm oil is added to the grated cassava mash resulting in a less sour and a pronounced yellow coloured gari.

Key informant interviews were held with extension officers in these states to assist in identifying key cassava producing, processing and marketing areas. Within those key areas, a total of 16 villages were randomly selected and village leaders were contacted to facilitate organisation of focus group discussions. Villages that were actively involved in cassava production and processing were identified, and two villages in each state were randomly selected as trial sites: Ilupeju (Iwo railroad station), Aiyedire Local Government Area (LGA), Agbora Bamgbola, Irewole LGA in Osun and Umunam Imerienwe, Ngor‐Okpala LGA and Obibiezena, Owerri North LGA in Imo.

### Design and management of mother and baby trials

#### Overview

**Figure 1:**
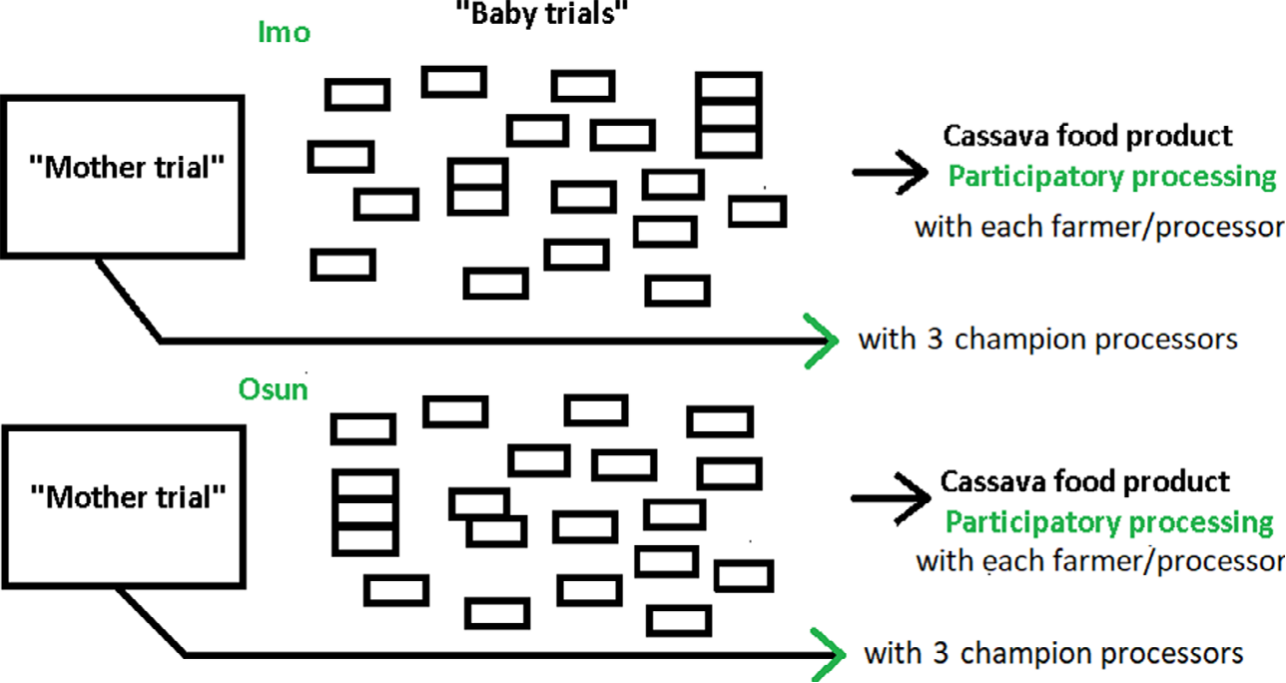
Trial set‐up including a mother trial and baby trials in each of two states (Osun and Imo state). Mother trials contained 20 and 23 varieties for Osun and Imo respectively. Each baby trial contained five varieties.

A *mother and baby trial* design makes it possible to acquire quantitative and qualitative data on variety performance. The main trials (*mother trials*) included all tested varieties and were planted in each of Imo and Osun States in a farmer’s field. The *baby trials* were subsets of the varieties in the mother trial, planted in farmers’ fields for the purpose of cross‐checking performance (Snapp, 2002; Snapp *et al*., 2002; Atlin *et al*., 2002). In Osun and Imo, one mother trial was installed in one of the two selected villages per state, and one baby trial was installed in each of 20 farmer–processor fields (not related to sites of the mother trials) spread out over the two villages per state (a total of two mother trials and 40 baby trials; Fig. 1).

#### Selection of baby trial participants

To motivate participation in field trials (e.g. Misiko, 2009, 2013; Van Etten *et al*., 2016), and to be socially inclusive, discussions were held with community leaders on the kind of social groups present among the smallholders in the community. We included participants in such a way that each local social group in which cassava growing and processing expertise existed was proportionally represented. To include agronomic as well as processing knowledge, we selected people with hands‐on experience in both cassava cultivation and processing of the crop into food products, that is farmer–processors. Through this expertise focussed approach, we ended up with all but one women participants. This strategy builds on earlier research that showed that preferred traits were similar for men and women, but women mentioned food product‐related traits such as quality of gari and fufu more often than men, while men mentioned agronomic traits more often, reflecting the gendered division of labour (Wossen *et al*., 2017; Teeken *et al*., 2018).

### Varieties tested

Varieties selected for evaluation were comprised of (i) varieties grown and appreciated by farmer–processors in each of the trial location’s communities; (ii) commonly grown landraces in the main cassava growing areas in Nigeria (based on Wossen *et al*. (2017)); and (iii) IITA/NRCRI improved varieties, including both older and more recent releases developed using a shortened breeding cycle made possible by using genomic selection (Table 1). All of these varieties were either landraces or improved varieties, and identities were verified using genetic fingerprinting following a single nucleotide polymorphisms (SNPs) protocol as described in Rabbi *et al*. (2014) and Wossen *et al*. (2017). Common local varieties encountered in the communities in each state were added to the main set of 15 varieties. Local varieties from one state were not taken to the other state.

**Table 1:** Cassava varieties evaluated in Osun and Imo State

Varieties (local name)	Type of variety		Variety group*	Location/type of trials			
				Imo		Osun	
				MT	BT	MT	BT
TMEB1 (Antiota)	Landrace, commonly grown, (released 1986)		1	X	X	X	X
TMEB2 (Odongbo)	Landrace, commonly grown, (released 1986)		1	X	X	X	X
TMEB419 (Breeder Check)	Landrace, commonly grown from Togo (released 2005)		1	X	X	X	X
TMEB7 (Oko Iyawo)	Landrace, commonly grown		1	X	X	X	X
TMEB693 (Ankara/Dehor)	Landrace, commonly grown in Ghana (boiling & pounding)		1	X	X	X	X
TMS‐IBA30572	Improved, commonly grown, IITA (released 1984)		2	X	X	X	X
WK195	Improved, commonly grown, IITA/NRCRI (1970s)		2	X	X	X	X
NR8082	Improved, commonly grown (released 1986)		2	X	X	X	X
TMS‐IBA980505	Improved IITA (released 2005)		2	X	X	X	X
TMS‐IBA980581	Improved IITA (released 2005)		2	X	X	X	X
TMS‐IBA010040	Improved IITA (released 2010)		2	X	X	X	X
TMS‐IBA961632	Improved IITA, commonly grown Osun (released 2006)		2	X	X	X	X
TMS13F1365P0002	Improved IITA/Nextgen (crossed 2013)		2	X	X	X	X
TMS13F1160P0004	Improved IITA/Nextgen (crossed 2013)		2	X	X	X	X
TMS13F1176P0002	Improved IITA/Nextgen (crossed 2013)		2	X	X	X	X
Chigazu	Landrace (not in library)		1	X			
Durungwo	Landrace (not in library)		1	X			
Kati Kati	Landrace (not in library)		1	X			
Mgboto Umuahia	Landrace		1	X			
Nwocha	Landrace (not in library)		1	X			
Salome (6 months)	Landrace (not in library)		1	X			
Agric	Improved (clusters with IITA‐TMS‐IBA030075. Crossed 2003)		2	X			
Nwageri	Improved (clusters with IITA‐TMS‐IBA090144. Crossed1996)		2	X			
Akpu	Landrace		1			X	
Omoh Local 2	Landrace (clusters with TMEB1 Released 1986)		1			X	
Honourable 1	Improved (clusters with IITA‐TMS‐IBA960304 Crossed 1996)		2			X	
Honourable 2	Improved (clusters with TMS‐IBA30572 Released 1984)		2			X	
Omoh Local 1	Improved (clusters with BDS_96_119/BDS_96_157. Crossed 1996)		2			X	
Cotonou	Landrace (clusters with TMEB419 Released 2005)		1				X
Fulani	Landrace (clusters with TMEB419 Released 2005)		1				X
Akpu	Landrace		1				X
Nikan Pupa	Landrace (clusters with TMEB1 Released 1986)		1				X
Atu	Landrace (clusters with TMEB7)		1				X
Oko Iyawo	Landrace (clusters with TMEB7)		1				X
Agric	Improved (clusters with TMS‐IBA30572 Released 1984)		2				X
IITA	Improved (clusters with TMS‐IBA30572 Released 1984)		2				X
White Cassava	Not assessed						X
Awolowo	Not assessed						X
Ada_Nwanko	Not assessed				X		

BT, baby trial; MT, mother trial.

* Variety groups are identified as follows: 1. landraces, 2. improved varieties.

In total, 20 and 23 varieties were included in Osun and Imo, respectively (Table 1).

#### Trial design and management

Mother trials were planted in 2017 and 2018 while baby trials were only planted in 2017. All fields were prepared using manually formed ridges. Stem cuttings of cassava were provided by the IITA cassava breeding programme from multilocation trials in Nigeria. Mother trials were planted in a completely randomised block design with three replications. Plot sizes were 5 m by 6 m containing thirty plants (spaced 1 m by 1 m). In line with farmers’ practices in the study areas, no fertiliser was used. Plots were hand‐weeded four times for the mother trials and 3–4 times for the baby trials.

For the baby trials, each participant grew a different random combination of three varieties of the total of 15 in the main set of varieties. The packages of three varieties were distributed in such a way that each variety was replicated five times among the twenty participants. Each of the twenty participants also planted a breeder’s check (TMEB419) and a landrace variety of their choice, making a total of five varieties per baby trail. Each participant, with support from project personnel, planted the five varieties in two replications. Plot size was the same as in the mother trials.

All trials were harvested after 12 months. For the mother trials, harvesting was done successively in four batches of five varieties in Osun (a total of twenty varieties), and three batches of five and two batches of four varieties in Imo (a total of twenty‐three varieties) to realistically manage the number of pairwise comparisons by farmer–processors. The varietal representation of each batch was determined by randomisation of all the varieties in each location. For all trials, twelve plants of each plot were harvested (excluding the border rows). For both mother and baby trials, roots of the different replications were bulked together prior to processing.

#### Selection of champion processors

The mother trials constituted the source of roots for the processing evaluation with three *champion processors*, that is farmer–processors in the community who were renowned for their excellence in processing cassava into gari/eba and fufu. Champion processors were determined in a meeting organised with a random selection of village residents. All champion processors were women. In Osun, these included immigrants (from the South‐South region in Nigeria) chosen by the majority of autochthone villagers. This allowed for, in addition to gari and eba, the preparation of two different kinds of fufu in Osun. One type is turned on the fire in a cooking pot (common in the Southwest). The other is boiled in a bain‐marie, pounded, again boiled in a bain‐marie and pounded again to get the final paste, resulting in a more dense and firm fufu as is common in the South‐South (Aburime *et al*., 2019). In Imo, no fufu was made.

### Root and food product quality evaluation

#### Gari/eba processing

The harvested roots from the mother trials were divided into three equal portions and were processed by the three champion processors. Each batch of varieties from the mother trials was processed into gari/eba. Within each state, the same locally preferred processing equipment and the same procedure were used to process all the varieties in line with the processors’ customs and expertise. Skilled processors tacitly adjust the processing procedure (e.g. time of toasting) as needed to account for unexpected conditions or variables (Ezeocha *et al*., 2019).

Following local practices, the processing of the gari started with peeling the roots the day after harvest. Each of the three champion processors processed all the varieties. Following local custom, grating was done using a stationary grater in a processing centre in Osun and a mobile grater in Imo. In Imo only, following local practice, 32 mL of palm oil was added per 10 kg of grated fresh roots.

Fresh root weight, peeled root weight and gari weight were measured. The gari yield was calculated based on the peeled root weight. The champion processors prepared eba as they are used to by mixing of gari with hot water and stirring until they obtained the desired texture.

**Figure 2:**
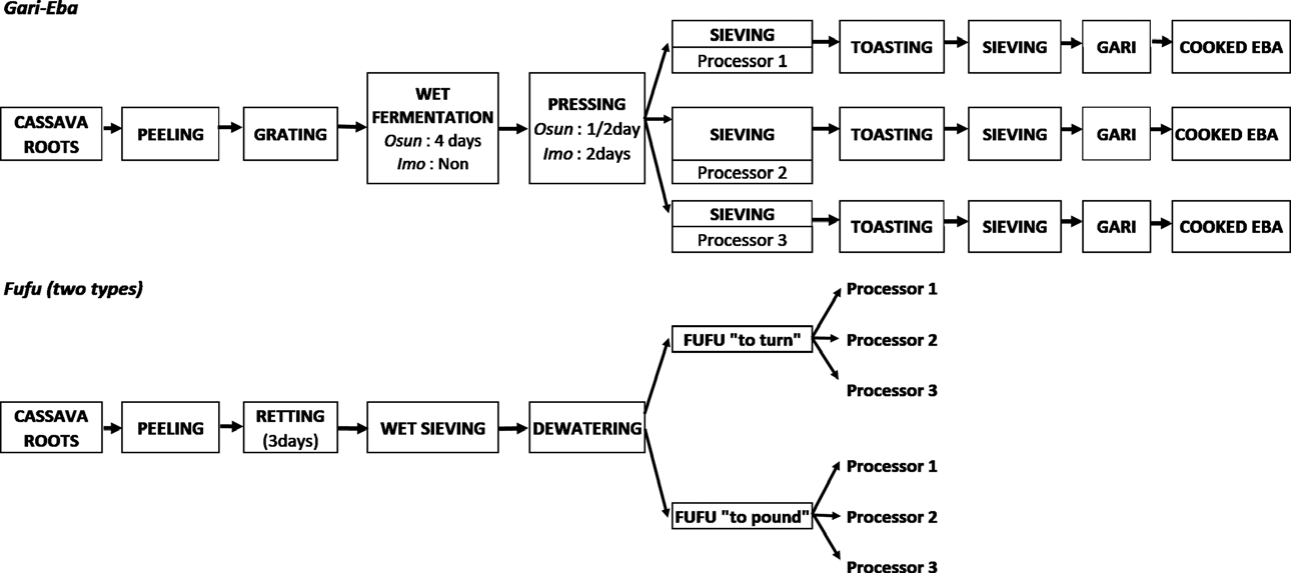
Processing flowsheet for*gari*in Osun and Imo state and for*eba*and two types of*fufu*in Osun state.

The baby trials were processed into gari by the farmer–processor trial participants, using their household’s preferred processing equipment. In Imo, in‐house processing was done using a mobile grater, toasting pans and small in‐house presses. In Osun, processing centres with stationary grating machines, large presses and toasting boards were used. Similar to the mother trials, the following quantities were measured during the processing: fresh root weight, peeled root weight and gari weight. Fig. 2 shows the detailed processing steps to obtain gari‐eba and fufu from the mother–baby trials.

#### Fufu processing

For the Osun mother trial, two types of fufu were prepared: turned and pounded. Fufu processing started with peeling and retting directly on the day of harvest following the local practices. Large plastic barrels were used to ret the roots for three days. The following quantities were measured during the processing: fresh root weight, peeled root weight and fufu weight. Fufu yields were calculated based on the peeled root weight.

#### Quality evaluation using pairwise ranking

Pairwise comparison and ranking (Russell, 1997; Catley *et al*., 2013 cited in Nduwumuremyi, *et al*., 2016) of varieties for overall quality of fresh roots and the subsequent food products from the processors was employed to enable tapping into the tacit knowledge of the farmer–processors and facilitate full participation by those with lower verbal and dialectic skills. The approach emanates from the notion that the stakeholders (farmer–processors) will be best able to evaluate varieties planted, harvested and processed in their own working environment (Richards, 1986; Millar, 1994, Ceccarelli *et al*., 2000, 2001; Ezeocha *et al*., 2019) in which different varieties are evaluated alongside each other (Van Etten, 2011; Van Etten *et al*., 2016). Champion processors evaluated the roots and food products from the batches (mother trial), and farmer–processor baby trial participants evaluated the roots and related food product from the five varieties in their own baby trial. Pairwise comparisons were made on the fresh roots and the food products (gari, eba and fufu). Fresh root ranking was done separately for gari and fufu: ‘Fresh roots for gari’ and ‘fresh roots for fufu’ champion processors and baby trial participants were motivated to interact with the roots and food products as they wished, for example by cutting, feeling, smelling and tasting. To avoid prejudging based on participant perceptions of prior samples, in the steps from fresh root to eba, samples were re‐labelled each time.

At each pairwise comparison (10 for a batch of five varieties, and six in the case of a batch of four), processors were asked to provide reasons for choosing one variety over the other, in order to elicit critical user quality criteria. In cases where certain varieties won equal times relative to others, those were again pairwise compared to obtain the final rank. No pairwise ranking on eba in Imo was done. Instead, the eba products in Imo were simply ranked from most to least preferred by the farmer–processors by observing all samples simultaneously.

#### Fresh root quality

Dry matter content was determined in the field using the specific gravity method comparing the root weights in water and in air (Kawano *et al*., 1987). Each measure was based on five roots of different sizes from each plot: one small, two medium and one large‐sized root.

#### Gari quality based on laboratory measurements

##### Physical colour

The physical colour in L*, a* and b* of the gari was measured using a Minolta CR‐ 400 Chroma Meter (Minolta Corp., Osaka, Japan). The browning index (BI), based on L*, a* and b* values, was determined using the method described by Pathare *et al*. (2013).

##### Starch and sugar

The total starch and sugar contents were determined using the AOAC method (2006). About 0.02 g finely ground gari sample was weighed into a clean centrifuge tube, wetted with 1 mL of ethanol and 2 mL of distilled water followed by 10 mL of hot ethanol. The mixture was vortexed and centrifuged using a Sorvall centrifuge (model GLC‐1; Ivan Sorvall Inc., Newtown, CT, USA) at 423 ***g***  for 10 min. Absorbance was read at a wavelength of 490 nm.

##### Total titratable acidity

The total titratable acidity of the samples was determined by titration with 0.01 N NaOH (AOAC, 1990). Values were expressed in equivalent grams acetic acid per 100 g of sample.

#### Functional properties of gari

The water absorption capacity (WAC; g mL^−1^) and dispersibility (%) of gari were determined using methods described by AOAC (2006). For WAC, 1 g of each gari sample was weighed into a 15 mL centrifuge tube with 10 mL of distilled water, centrifuged at 512 ***g***  for 15 min, and the weight of sediment was determined.

Dispersibility was determined by weighing 10 g of the sample into a 100‐mL measuring cylinder and distilled water added to reach a volume of 50 mL. The mixture was stirred vigorously, particles were allowed to settle for three hours and the percentage dispersibility was calculated.

Bulk density (g mL^−1^) was determined using the method reported by Abiodun *et al*. (2010). The swelling power (SP; g mL^−1^) and solubility index (%) were determined using the method described by Riley *et al*. (2006) with a slight modification, where 50 mL of distilled water was added to 1 g of the sample in a centrifuge tube and incubated for 30 min in a water bath at 95 °C. The mixture was centrifuged at 512 g  for 15 min, and the mass of soluble substances in the supernatants was calculated by difference with the mass of the sediment.

### Data analysis

Pairwise ranking results of varieties in each batch grown in the mother trials of both years were combined. As the content of each batch was newly randomised each year, the second year provided additional combinations for comparison. Data for Osun and Imo were analysed separately. Correspondence principal component analysis (PCA) of the ranks per batch as provided by farmer–processors was done using the PROC CORRESP procedure in SAS 9.4, Cary, NC, USA. This analysis was combined with the Bradley–Terry model analysis (Bradley & Terry, 1952; Van Etten *et al*., 2016) of all the individual pairwise ranks to obtain estimates of the chance that a variety is preferred over others. PCA of colour, and gari functional and physico‐chemical properties related to the varieties and their derived gari were done using R statistics software.

Root and food product quality characteristics preferred by processors were determined by coding all the reasons given at each pairwise comparison based on the mother and baby trials. Frequencies of each code were a proxy for the relative importance of each characteristic. Frequencies related to the mother and baby trials were analysed separately to reflect the champion processors’ and baby trial farmer–processor participants’ criteria.

## Results and discussion

### Genetic fingerprinting of varieties sourced from the communities

Most of the farmer varieties sourced from the communities of the trial locations were landraces and some of them clustered with released landraces TMEB419 and TMEB1 or the unreleased commonly grown landrace TMEB7 (Table 1). Only two varieties clustered with the same commonly grown improved variety TMS‐IBA30572, while four varieties clustered with improved unreleased materials. This is in line with the finding of Thiele *et al*. (2020) that unreleased varieties are prominent among the improved varieties cultivated by farmers.

### General agronomic performance of trials

From a food science perspective, a good quality root for gari should be harvested at maturity (ranging from about 8 to 18 months after planting, depending on the variety) and have high dry matter content. The higher the moisture content of cassava roots, the lower the gari yield, the longer the roasting time and the lower the gari final quality (Abass *et al*., 2017). However, processors are known to be able to adjust procedures to attain high quality of gari despite differences in root dry matter (Ezeocha *et al*., 2019; Dahdouh *et al*., 2020). Cassava roots with high fibre content are typically less suitable for gari production (Mkumbira *et al*., 2007). Overall, user preferences indicate that a good quality root for gari and fufu production was harvested at maturity, is medium to large in size, has high density and low water content, has a white bright colour, has smooth outer skin, and is not rotten and does not have foamy parts inside (Chijioke *et al*., 2020; Ndjouenkeu *et al*., 2020).

Table S1a,b show the general yield‐related performance of the mother trials, including the food product yield. Yield data are in line with results from breeders’ trials: Fresh and dry root yield, were higher for improved varieties. There was no difference in root dry matter. In Osun, product yield of gari was also higher for improved varieties (7.15 and 6.92 kg per 20 kg of peeled roots, *t*‐test, *P* = 0.096). However, the fufu yield *pounded* in Osun was higher for landraces (6.22 and 5.44 kg per 20 kg of fresh roots, *t*‐test, *P* = 0.064). The baby trials in Imo only showed a significant difference for dry matter. The landraces had a higher average (33.7% and 32.5%, *t*‐test, *P* = 0.034). In Osun, the same trend was observed for dry matter with an even higher difference: 37.05% and 34.8 % (*t*‐test, *P* = 0.0001) while the gari yield was also significantly higher (7.2 and 6.5 kg of peeled roots, *t*‐test, *P* = 0.015) for landraces (Table S2). This indicates that on‐station breeding trials and the mother trials in farmers’ fields show similar patterns in agronomic performance, while the combined baby trials in 20 different farmers’ fields in each state showed the need for testing breeding materials and new varieties under real farmer management and agronomic conditions.

These findings indicate that increasing dry matter content together with fresh yield will increase gari product yield for each unit of harvested cassava, which is broadly in line with farmers’ and processors’ priorities. The result for fufu, however, indicates a possible trade‐off between fresh yield and fufu yield, which might indicate product loss because of higher fibre content or more probably because of incomplete softening in the fresh roots of improved varieties during retting. However, product yield must go along with a good quality food product which is of very high importance to cassava farmers and processors (Awoyale *et al*., 2015; Wossen *et al*., 2017; Teeken *et al*., 2018; Olaosebikan, *et al*., 2019).

### Food product quality evaluation by farmer–processors

**Figure 3:**
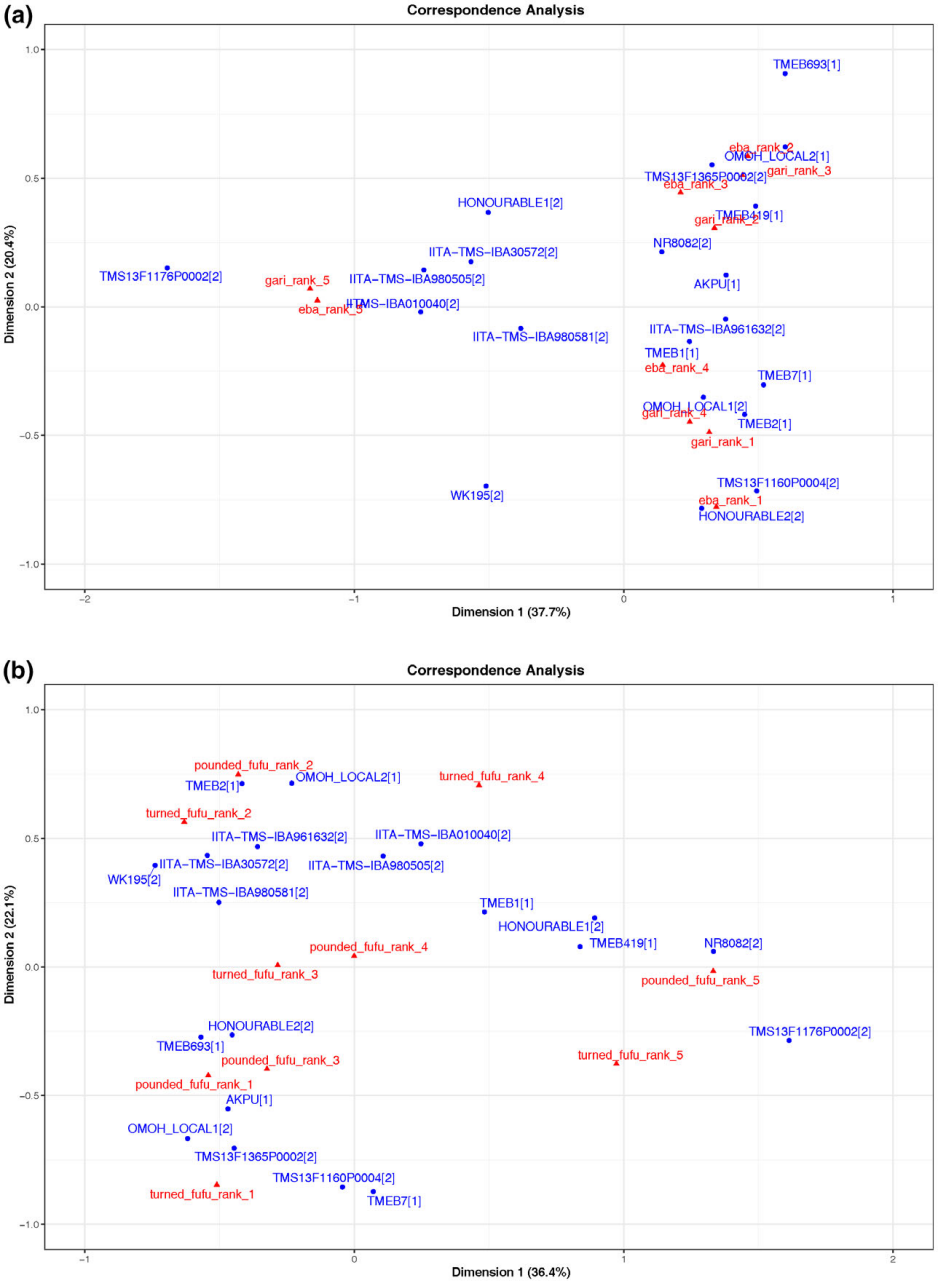
Correspondence PCA based on processor ranking of*gari*(gari_rank) and*eba*(*eba*_rank) in Osun state based on two‐year data from the mother trials (a) and turned*fufu*(turned _*fufu*_rank) and pounded*fufu*(pounded*fufu*_ rank) (b). The numbers 1–5 indicate the rank (1 = best quality, 5 = lowest quality). Numbers behind variety names indicate the variety groups: 1. Landraces 2. Improved varieties.

**Figure 4:**
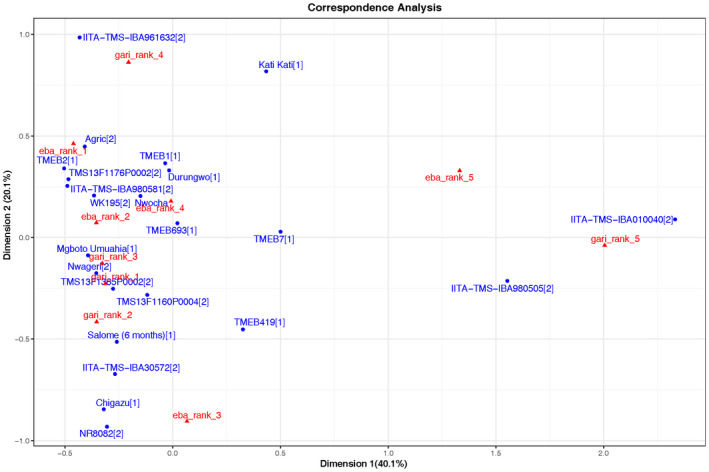
Correspondence PCA based on processor ranking of*gari*(*gari*_rank) and*eba*(*eba*_rank) in Imo state based on two‐year data from the mother trials. The numbers 1–5 indicate the rank (1 = best quality, 5 = lowest quality). Numbers behind variety names indicate the variety groups: 1. Landraces, 2. Improved varieties.

**Figure 5:**
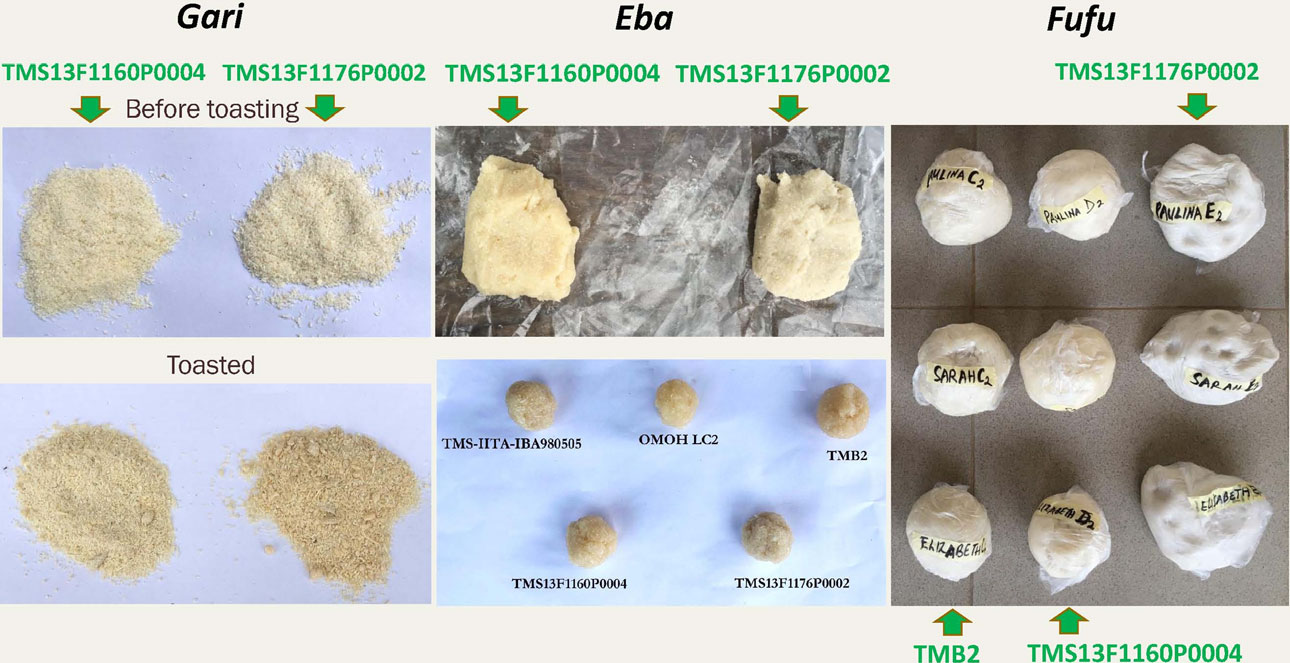
Observed colour issue with regard to variety TMS13F1176P0002 in the trials in Osun state, in untoasted mash before toastin,*gari, eba*and fufu (brownish darker or grey colour compared to other varieties) and texture issue in*fufu*(not holding shape and sacking out and being too soft). Photographs relate to the food products produced from the mother trial.

Table 2 shows the Bradley–Terry estimates for the champion processors’ evaluation of fresh roots for gari, and the food products gari and eba, as well as fresh roots for fufu, and the food products fufu pounded and fufu turned in Osun. Table 3 shows the estimates for fresh roots for gari and for gari in Imo. Higher Bradley–Terry estimates indicate that a variety is more preferred. The best five varieties (higher values) and the worst five varieties (lower values) are highlighted in green and red, respectively. Results for Osun from both the Bradley–Terry and correspondence PCA analyses (Figs 3 and 4) show that the landraces TMEB693 and Akpu rank first in food product quality for gari and eba, respectively (*P* < 0.00 and *P* < 0.05, respectively). At the same time, the five lowest‐ranking varieties for both gari and eba are all improved varieties except for TMEB2. For fufu, however, the improved varieties IITA‐TMS‐IBA980581 (*P* < 0.05) and TMS13F1160P0004 (not significant but very high estimate) and Honourable 2 (*P* < 0.05) rank first for pounded and turned fufu, with mostly improved varieties among the five lowest evaluated varieties. In Imo, a similar trend for the gari product was observed, where the local landrace Mgboto_Umuahia is the clear winner (*P* < 0.01) followed by the landrace Salome (6 months; *P* < 0.01) and the improved variety Nwageri (*P* < 0.01). The improved varieties TMS13F1160P0004 and TMS13F1365P0002 are also among the higher‐ranked varieties (*P* < 0.05 and *P* < 0.01, respectively). With regard to the lowest‐ranked varieties, we see a mixture of landraces and improved material with the improved IITA‐TMS‐IBA980505 and IITA‐TMS‐IBA010040 ranked low for the gari product, just as in Osun. These two varieties are also very clearly clustering in a group of their own, with the lowest ranks for gari in Imo (Fig. 4) but also for gari and fufu in Osun (Fig. 3a,b). Overall, TMS13F1160P0004 ranked well for all the food products in both locations. Another clear result is that in Osun, the variety TMS13F1170P0002 clusters away in both the correspondence PCA for gari and fufu, confirming the observed colour and texture differences with this variety by processors (Fig. 5). The overall low Bradley–Terry estimate for all the food products in Osun for this variety confirms this. However, in Imo, where palm oil is added to the gari and fermentation time is much less than in Osun, TMS13F1170P0002 performs rather well. The large variation in the data, where well‐rated varieties for gari can be lower ranked for eba or fufu and vice versa, can be explained by the large influence of food processing factors. However, the observed relationship between food quality and variety supports the argument to include food product quality evaluation within evaluation of breeding trials.

**Table 2:** Bradley–Terry analysis of the evaluation of fresh roots and food product by ‘champion processors’, based on two‐year data from mother trial in Osun State

Category/variety	Group	Fresh roots for gari	*Gari*	*Eba*	Fresh roots for *fufu*	*Fufu* pounded	*Fufu* turned
Local							
AKPU	1	**−0.26**	1.87****	0.97**	−1.87****	−1.19**	**0.14**
HONOURABLE1	2	**0.06**	0.21	0.32	−0.84*	−1.58***	−2.29****
HONOURABLE2	2	−0.8	2.05****	0.17	**0.37**	−1.09**	1.37**
OMOH_LOCAL1	1	**0.62**	0.8*	**0.54**	−1.58***	−0.67	**0.14**
OMOH_LOCAL2	2	−1.34**	1.13**	0.19	−1.25**	−1.20**	−1.092
Common landraces							
TMEB1	1	−1.56***	0.78*	−0.06	−0.99**	−0.53	−0.49
TMEB2	1	−1.53***	0.25	**−0.83**	−1.66***	−0.32	−0.65
TMEB419	1	−3.03****	0.89**	−0.46	−1.29**	−1.44**	−1.27*
TMEB693	1	−2.07****	2.24****	0.25	−1.13**	**0.63**	−1.27*
TMEB7	1	−2.03****	0.95**	0.77*	−2.36****	**0.69**	**−16.55**
IITA/NRCRI Improved							
IITA‐TMS‐IBA010040	2	−1.57***	**−0.57**	−1.39***	**−0.50**	−1.16**	−2.46****
IITA‐TMS‐IBA30572	2	−1.61***	**−0.03**	−1.13**	−2.19****	−0.01	−0.066
IITA‐TMS‐IBA961632	2	−1.57***	2.04****	**0.42**	**0.27**	−0.44	−0.8
IITA‐TMS‐IBA980505	2	−1.99****	−1.65***	−1.78***	−1.25**	−1.20**	−3.98****
IITA‐TMS‐IBA980581	2	−3.21***	0.59	−0.46	−2.88****	1.51**	−1.2688*
NR8082	2	−1.40***	0.48	0.3	−1.10**	−1.99****	−2.38****
TMS13F1160P0004	2	−1.58***	0.79	**0.65**	**0.41**	−0.45	**13.49**
TMS13F1176P0002	2	−2.41***	−1.47**	−4.52****	−3.34****	**−15.94**	−1.59**
TMS13F1365P0002	2	**−0.38**	0.65	−0.53	**0.00**	**0.00**	**0.00**
WK195	2	**0.00**	**0.00**	0.00	−1.44***	**1.00**	−1.08*

Varieties with higher estimates are preferred more often in paired comparisons than those with lower estimates. Results from the best five and worst five varieties are highlighted in bold green and bold red, respectively, for *gari*, *eba*, ‘pounded’ fufu and ‘turned’ *fufu*. The groups represent the landraces (i) and improved varieties (ii). Significance levels indicate the probability that the estimate is significantly different from 0: *P < 0.10; **P < 0.05, ***P < 0.010, ****P < 0.001.

**Table 3:** Bradley–Terry analysis of the evaluation of fresh roots and *gari* by ‘champion processors’, based on two‐year data from mother trial in Imo State

Group/variety	Group	Fresh roots for gari	Gari
Local			
Agric	2	−12.09	0.09
Chigazu	1	**−14.94**	1.36
Durungwo	1	−11.92	0.81
Kati Kati	1	**−12.71**	−1.28**
Mgboto_Umuahia	1	−11.97	2.03***
Nwageri	2	−12.43	1.86***
Nwocha	1	**−10.90**	0.03
Salome(6 months)	1	−12.53	2.02***
Common landraces			
TMEB1	1	**−10.40**	‐0.08
TMEB2	1	**−10.10**	0.39
TMEB419	1	**−10.84**	−0.90*
TMEB693	1	−11.90	0.82
TMEB7	1	−12.11	**−0.45**
WK195	1	−12.38	0.00
IITA/NRCRI improved			
	2	−12.30	**−14.97**
IITA‐TMS‐IBA30572	2	−11.81	0.42
IITA‐TMS‐IBA961632	2	**−9.37**	0.05
IITA‐TMS‐IBA980505	2	**−13.59**	−1.25*
IITA‐TMS‐IBA980581	2	−12.20	1.26*
NR8082	2	**−12.80**	1.67**
TMS13F1160P0004	2	**−25.63**	1.62**
TMS13F1176P0002	2	−11.75	1.03
TMS13F1365P0002	2	−11.11	1.58***

Varieties with higher estimates are preferred more often in paired comparisons than those with lower estimates. Results from the best five and worst five varieties are highlighted in bold green and bold red, respectively, for *gari*. The groups represent the landraces (i) and Improved varieties (ii). Significance levels indicate the probability that the estimate is significantly different from 0: *P < 0.10; **P < 0.05, ***P < 0.010, ****P < 0.001.

The Bradley–Terry results show that fresh roots were ranked relatively similarly. However, in the evaluation of fresh roots to be processed into fufu in Osun, there is a clear set of varieties that are not preferred: Akpu, IITA‐TMS‐IBA30572, TMEB7, IITA‐TMS‐IBA980581 and TMS13F1176P0002. These varieties all perform better when evaluated for the fufu product itself. TMS13F1176P0002, however, stays among the lowest‐ranked and TMEB7 and IITA‐TMS‐IBA980581 are among the highest, especially for the pounded fufu. The smaller distinction in the fresh root ranks can be understood methodologically: in the last breeding stage (on‐farm trials), fresh roots are often evaluated cooperatively between farmers and breeders, resulting in breeders having good knowledge about the type of fresh roots preferred by farmers. Importantly, this indicates that users cannot predict the food product quality based only on testing the fresh roots, which explains the limited feedback on food product quality through participatory variety selection (PVS) trials. Local varieties and landraces are dominant among those most preferred for gari‐eba food quality. However, the clear presence of some recently improved varieties like TMS13F1160P0004 (in both locations) and TMS13F1365P0002 in Imo is encouraging for breeders. For fufu, some improved varieties seem relatively more suitable, stressing the need to keep both product profiles in view.

The preferred quality of the food product of landraces Mgboto_Umuahia, TMEB693 and Akpu needs follow‐up and further evaluation by breeders for possible release or for use as parents for improved breeding lines. This also counts for the older improved varieties like Nwageri (genetically clustering with IITA‐TMS‐IBA090144) and Honourable 2 (genetically clustering with IITA‐TMS‐IBA30572). However, Honourable 2 was ranked very differently from IITA‐TMS‐IBA30572. IITA‐TMES‐IBA30572 is also well‐known among agricultural extension services for its good food product quality (Tokula & Ekwe, 2006).

There is also a clear difference between Osun and Imo with regard to varieties liked and not liked for gari quality. This can be related to the different fermentation times practised as well as to the addition of oil to the grated mash before fermentation in Imo. A more unfavourable appearance can be hidden by the yellow oil colour, and other quality issues might be important when less fermentation is practised. As the processing of cassava into white gari (without added palm oil) is also a common practice in Imo, results for the Imo location in this study are representative only for the yellow gari product.

While the Bradley–Terry estimates for the baby trial results (Tables S3a,b) show more random variation compared to the mother trials, they also show the dominance of landraces among the best ranked for food quality. Nikan Pupa and Fulani (both genetically clustering with landrace TMEB419) are among the best landraces for Osun, while Mgboto_Umuahia is among the best for Imo. Again confirmed is the excellent performance of TMS13F1160P0004 both for gari and eba quality in both Imo and Osun, as well as the low ranking for IITA‐TMS‐IBA980581, IITA‐TMS‐IBA010040 and TMS13F1176P0002 in both locations. Similar tendencies observed for mother and baby trials confirm the relevance of these findings, especially when considering the diversity of processing methods and environments tested.

### Elicited quality descriptors

A review by Béchoff *et al*. (2018) citing Nigerian studies by Achinewhu *et al*. (1998); Irtwange & Achimba (2009) and Owuamanam *et al*. (2010) shows that gari is judged by its swelling capacity, colour (bright), particle size (homogenous), moisture content (dry) and degree of sourness. Recent research on user preferences of gari in Nigeria and Cameroon show that the main quality aspects of a good gari are as follows: dry, white, shiny/attractive appearance, sourness or sweetness according to local tastes, heavy (dense) and uniform granule size. A good eba is characterised as smooth, firm, sticky, elastic, mouldable and having good swelling of the gari during preparation of eba (Ndjouenkeu *et al*., 2020). A good fufu paste is associated with softness, shiny cream colour (Tomlins *et al*., 2007), well mouldable into balls, only a little sticky, stretchable and bland or distinct fermented odour depending on local preferences (Béchoff *et al*., 2018).

Table 4 shows the important descriptors elicited for fresh roots, gari/eba and fufu by champion processors and farmer processors for each of Osun and Imo during the pairwise rankings. For the fresh roots descriptors, breeder selection of high dry matter (which combines low water content, heavy/dense roots and a high food product yield) is in line with user demand. However, several more and equally important characteristics are mentioned such as colour, ease of peeling, large‐sized roots (which can be partly related to ease of peeling, i.e. larger roots have a smaller total surface per unit of weight), swelling of the food products when preparing them, and the very important softening of the roots during soaking for fufu. If roots do not get soft, or get only partly soft, large parts of the roots will get sieved out, resulting in reduced product yield. Softening would not have been elicited if fufu was not part of this study.

**Table 4:** Preferred characteristics of fresh roots and food products and their frequencies, elicited during pairwise ranking of fresh roots, *gari, eba and fufu* by champion processors (CP) and farmer–processors (FP) in Osun and Imo States, Nigeria

Fresh roots/state	Freq. %		Product	Freq. %		Product	Freq. %	
	CP	FP		CP	FP		CP	FP
Osun State								
*Fresh roots for Gari*			*Gari*			*Eba*		
High *gari* yield	68	37	Colour (white/ butter/not brown)	73	76	Good Appearance	86	68
High root yield	8	38	Heavy/dense	52	61	Heavy	48	15
Heavy/dense	43	28	Smooth	52	61	Smooth	41	30
Low water content	43	50	Good taste	35	38	Stretchable	29	28
Easy to peel	24	13	Crunchy	28	20	Firm	20	34
Colour (bright white)	22	17	Swells well making eba	23	8	Good taste	18	24
Large size	18	37	Good appearance	22		Swell well when prepared	15	2
Swelling while toasting	13	2	Neat (clean)	22	17	Neatness (clean)	14	19
No constrictions	8	14	High Yield	2		Mouldable	8	22
Smooth skin	4					Soft	7	
Poundable	1	3				Aroma	5	4
						Preservable	5	
						Starchy		14
*Fresh roots for fufu*			*Fufu turning*			*Fufu pounding*		
High fufu yield	65		Bright white/cream colour	64		Bright white/cream colour	68	
Heavy/dense	39		Heavy/dense	47		Heavy/dense	55	
Easy to peel	28		Firm	37		Firm	38	
Low water content	20		Mouldable	29		Smooth	30	
Colour (white)	19		Smooth	26		Swell well when cooking	28	
Large size	14		Preservable	26		Preservable	27	
Softening when soaked	12		Stretchable	20		Mouldable	16	
Swelling when cooked	11		Swelling when cooking	16		Stretchable	14	
No constrictions	7		Neat (clean)	5		High fufu yield	9	
Less fibrous	6		Good taste	5		Neat (clean)	8	
Stretchable	4		Soft	4		Good taste	7	
Smooth skin	3		Odourless	3		Soft	4	
Poundable	3		Non‐sticky (to hands)	3		Odourless	4	
Imo State								
*Fresh roots for gari*			*Gari*			*Eba*		
High *gari* yield	47	33	Bright	38	55	Good appearance	37	
High root yield	41	49	Good uniform granule size	32	3	Non‐sticky (to hands)	28	
Large size	38	53	Fine appearance	29	1	Stretchable	25	
Low water content	33	13	Swelling when making eba	29	13	Soft	20	
Easy to peel	1	4	Dry	27	16	Swell well when prepared	15	
Dry skin	8		Should accept oil	16	1	Heaviness	14	
*Gari* quality	7		High *gari* yield	15	14	Sticky	14	
Smooth skin	5		Sell fast	10		Firm	14	
Brighter *gari*	3		Should provide good *eba*	8		Bright	9	
Heavy/dense	2	10	No lumps	4		No dull colour	9	
Fine appearance	1		Heavy *gari* (dense)	3	44	High eba yield	9	
Darker skin colour	1		Good taste		10	Starchy	5	
White roots	1	6	Preservable		2	Smoothness	4	
Swell when toasted to gari		3						
No constrictions		2						

In summary, the most important preferred characteristics of gari are as follows: colour, brightness, appearance, uniform granule size of gari (smoothness) and heaviness (bulk density), which aligns with the literature references provided earlier that define a good gari. Bulk density of the gari is not necessarily related to dry matter because, during processing, water is removed. For gari and fufu, colour, brightness and appearance are by far the most important indicators of quality. In addition, for fufu, preservability and for fufu and eba textural properties such as firmness (cohesiveness), mouldability, stretchability (elasticity) and softness are very important and preferred at different levels depending on the region, culture and personal preferences (Béchoff *et al*., 2018; Adinsi *et al*., 2019; Ndjouenkeu *et al*., 2020). One major overall characteristic that appears important for eba and fufu is the swelling of the food product when prepared. Preferences for both types of fufu are identical but the degree to which each characteristic is preferred varies for the two fufu types, as pounded fufu is more elastic and harder than turned fufu. In Imo, brightness and appearance are stressed but not colour. This can be understood by the oil that is added by the processors in Imo, turning all the gari into a pronounced yellow colour. This practice is highlighted by the preferred characteristic in Imo of ‘should accept oil’, which is a measure of how well the variety takes the desired oily yellow colour. This characteristic was also identified by Ndjouenkeu *et al*. (2020).

Both in Osun and Imo processors mentioned that butter/yellow colours are preferred as long as they are bright and not brown or dark. A brown, dark or dull coloured gari or eba was absolutely disliked. When comparing the champion processors and farmer–processor baby trial participants, the latter put more stress on the heaviness (bulk density) of the gari. Table 5 sums up the most important quality descriptors of the fresh roots and the food products.

**Table 5:** Summary of crucial fresh roots and food product characteristics based on characteristics elicited from farmer–processors during pairwise ranking of roots and food products

Fresh roots	Gari	Eba	Fufu
High dry matter (low water content, heavy)	Bright colour	Good appearance (not dark in colour	White cream colour
High product yield	Uniform granule size	Heavy/Dense	Heavy/Dense
High root yield	Swelling when making *eba*	Smooth (good merging granules, no lumps)	Smooth (no lumps)
Large size	Chruchy/Dry	Stretchable (elastic)	Firm
Easy to peel	Heaviness (high bulk density)	Mouldable Not sticky (to hands)	Mouldable
Bright white	Good taste	Firm	Preservable
Softening when retted (*fufu*)	Oil acceptance (providing desired colour ‐if oil added‐)	Good taste	Swelling when cooked
			Stretchable (elastic)

As food product colour is also variety related (Akely *et al*., 2020), it is a potential target for improvement by breeders. Heritability determined for gari and fufu colour at IITA confirms this potential. Heritabilities for L*, a*and b* values range from 0.24 to 0.98 (unpublished data).

### Analysis of gari laboratory parameters and the link to quality assessment of the champion farmer–processors

Generally, a good quality gari has a maximal average moisture content of 10% (Codex, 1989; Sanni *et al*., 2005). The recommended starch content in gari is 75% while the fibre content should not exceed 2% (Sanni *et al*., 2005). Good quality gari should swell well when soaked in water (Almazan *et al*., 1987). If the bulk density is low, the gari will float on top of water and will not soak well and can be rejected by consumers (Sanni *et al*., 2008). A good gari is white, cream or yellow. The uniformity and brightness of the colour, however, are often more important than the colour itself. Concerning texture, gari with a smooth homogeneous texture is preferred. Gari must be crispy and should not contain sand particles, black specks or residual peels (Abass *et al*., 2017).

Tables S4a,b and S5a,b show the results of the analysis of the functional properties and physical colour together with the chemical properties. The analysis of variance for the data from Osun and Imo show a significant varietal effect. In comparison with improved varieties and landraces for all the measured parameters on the gari in Osun, only swelling power was higher for the landraces (6.88 and 6.62 *t*‐test, *P* = 0.099). Swelling power is an important aspect that can be related to the swelling of gari into eba which is, as we have seen, highly preferred by processors. For Imo, improved varieties had a higher starch content (82.14 and 80.89, *t*‐test, *P* = 0.076), but a lower b (yellow) value (28.68 and 29.75, *t*‐test, *P* = 0.018). The latter could indicate lesser oil acceptance in improved varieties. The higher starch content can contribute positively to the texture (firmness, cohesiveness) of eba (Akingbala *et al*., 2005).

To relate the champion farmer–processors’ evaluation to the laboratory analysis of the gari as well as the yield‐related data, data were compared using *t*‐tests for the best three and the worst three ranked varieties for gari quality. For Osun, swelling power (SP; 7.25 and 6.49, *P* = 0.025), total titratable acid (TTA; 0.66 and 0.56, *P* = 0.00) and yellowness (b*; 25.17 and 20.67, *P* = 0.050) were higher for the best evaluated varieties while water absorption capacity (WAC) (465.86 and 487.95, *P* = 0.055), bulk density (0.53 and 0.56, *P* = 0.020), solubility (6.35 and 6.83, *P* = 0.031) and redness (a*; 1.85 and 2.50, *P* = 0.022) were all significantly lower for the best varieties. For the other parameters, there were no differences. Farmer–processors prefer high swelling (expansion in volume) when cooking gari. It should be noted that swelling power in this study was determined using hot water, which is different from the often‐calculated swelling index using cold water (Sanni *et al*., 2008). Cold water results in a slower process of rehydration and does not include the gelatinisation of gari particles that were not fully gelatinised during toasting. Teeken *et al*. (2019) also found no correlation between the amount of gari swelling when making eba (expansion in volume of the eba) and the amount of swelling when mixing gari with cold water following the method of Sanni *et al*. (2008). This indicates that the highly appreciated swelling of gari while making eba cannot be evaluated by assessing only the swelling in cold water. The solubility index of gari produced from varieties in Osun ranged from 6.22% in TMS13F1160P0004 to 7.97% in Honourable 1 (Table S4a). Considering the preference for low solubility, TMS13F1160P0004 was indeed preferred for gari (although not among the top five) and eba. Apea‐Bah *et al*. (2011) reported that starchy foods with high solubility might result in a more moist and less cohesive dough, in contrast to preferences of the farmer–processors for firm mouldable dough. The solubility value for the variety TMS13F1176P0002 is among the higher values (7.06), explaining the low food product quality ranking for the eba for this variety (Table 2) and possibly also explains the low quality fufu ranking and the textural issue observed in fufu made from this variety (Fig. 5). The ranking of TMEB693 as the best for gari in Osun may be because of its low solubility (6.29; similar to TMS13F1160P0004), which in turn may be related to good textural properties for eba (Table 2). However, the low ranked variety TMEB7 also shows similar low solubility values, which indicates the complexity of parameters that determine preference.

When comparing the best and worst varieties for gari in Imo, we see that only dispersibility (32.22 and 27.39, *P* = 0.0022) and bulk density (0.62 and 0.58, *P* = 0.00) are significantly higher for the best varieties while WAC (444.61 and 488.32, *P* = 0.000), sugar (3.68 and 4.17, *P* = 0.000), TTA (0.21 and 0.35, *P* = 0.000) and a* (−1.14 and − 0.51, *P* = 0.0017) are all significantly lower for the best varieties. The other parameters showed no significant difference. The ranking of the Mgboto_Umuahia landrace variety as best for gari in Imo State may be attributed to its high bulk density. This counts also for preference for Nwageri, Salome and NR8082, which all are in the higher bulk density range.

The higher the dispersibility, the better gari reconstitutes in water (Kulkarni *et al*., 1991). This implies that in Imo, where a relatively more elastic eba is prepared and where gari granules are more rehydrated and merged into a solid mass, gari with a higher solubility is preferred to produce a more elastic eba.

Although water absorption is considered an important property for most starchy foods and a function of smaller granule size resulting in higher solubility (Tian *et al*., 1991; Udoro *et al*., 2014) higher WAC values for *gari* seem to be non‐preferred and possibly an indication of lack of the desired firmness: Gari granules are expected to keep some resistance in the mouth when chewed (Ndjouenkeu *et al*., 2020), which can also be related to increased solubility, thereby creating a lack of the preferred mouldable texture of eba.

The comparison between the best and worst varieties shows that champion processors in both Osun and Imo preferred varieties that have a lower redness (a* value) while brightness (L*value) is not different between the best and the worst varieties. These findings contrast with the observation by Abass *et al*. (2017) that often the brightness is more important than the colour. Given the large emphasis for a bright non‐brown colour by farmer–processors, this preference suggests that they look for gari with lower a* values. The browning index (BI), calculated based on the L*a*b* values, showed an identical level of significance as the b* value. The overall Pearson correlation of BI and a* in Osun and Imo was 0.99 for both states, which indicates that a* values are a proxy for browning in gari.

In Osun, gari yield was significantly lower for the best varieties for food quality compared to the worst (6.54 and 7.29 kg gari per 20 kg of fresh roots, *P* = 0.059), which indicates that gari yield is negatively correlated with the food quality. For Imo, fresh yield (21.42 and 15.68 tons ha^−1^, *P* = 0.093), dry yield (6.82 and 4.83, *P* = 0.076) and gari yield (7.57 and 6.03, *P* = 0.00) are all higher for the best varieties for quality, indicating that food product quality can be successfully combined with high yield.

Given the importance of bright colour among the quality criteria and the non‐preference of dark or brown colour, we looked at the a* values for the different varieties in more detail. This confirms the non‐preferred colour issue of TMS13F1176P0002 (Fig. 5), because it shows the highest a* value (3.16) among varieties in Osun (Table S5a). However, browning for TMS13F1176P0002 was not observed in Imo where its a* values were not high and its ranking for gari was higher. This might be ascribed to fermentation procedure, as well as the interaction with palm oil addition, a common practice in Imo. In Imo, the commonly grown farmer variety TMEB1 shows the highest a* value (0.20), which also corresponds with the low ranking of this variety for gari.

Browning/darkening after cooking can be related to chlorogenic acid (Adams & Brown, 2007; Wang‐Pruski & Nowak, 2007) and browning/darkening before cooking can be caused by enzymatic browning related to polyphenols (Friedman, 1997; Tomás‐Barberán & Espín, 2001) in the roots of other crops such as potato. Because Fig. 5 shows that the non‐preferred colour of TMS13F1176P0002 already appears in the fermented mash just before gari toasting, it might be related to polyphenols in the cassava. This needs further investigation.

### Correlations among the functional and chemical properties, the physical colour of gari and the yield‐related parameters

Table S6a,b provides the Pearson correlations between the laboratory measurements on gari, gari yield and yield‐related fresh root parameters. There is no relation between the root dry matter and the bulk density of the gari, confirming that a high bulk density gari can be obtained from a low dry matter root. Gari yield, however, is correlated with root dry matter content in Imo (*r* = 0.72, *P* < 0.01) but not in Osun. Furthermore, root dry matter in Imo is positively correlated with dispersibility (*r* = 0.57, *P* < 0.01) and brightness (*r* = 0.58, *P* < 0.01), both preferred by farmer–processors in Imo. In Osun, root dry matter is positively related only to solubility (*r* = 0.61, *P* < 0.01) which as we have seen is significantly lower for the best‐ranked varieties. Focussing on high dry matter under longer fermentation conditions could negatively influence the firmness of the eba produced.

There is a negative relation between the redness (a*) and the sugar content. In the longer fermentation environment of Osun, higher sugar content results in lower values of a* (*r* = −0.76, *P* < 0.01) and higher b* values (*r* = 0.57, *P* < 0.01). In Osun, the TTA, a measure of the acidity of the gari (Béchoff *et al*., 2018), is negatively related to the sugar (the more sugar that is used by the lactic acid bacteria during fermentation, the more sour the gari gets). Consequently, the more acid the gari, the less sugar it contains, which is to the detriment of the bulk density (the relation between bulk density and TTA is negative: *r* = −0.60, *P* < 0.010) and increases browning (a* values). While sugar and browning are correlated, further study is needed in order for breeders to use these traits in high throughput screening.

For the Imo situation, with less fermentation time, the relation between a* and sugar is reversed, and higher values for sugar are correlated with higher a* values (*r* = 0.83, *P* < 0.01) and higher b* values (*r* = 0.69, *P* < 0.01). The relation between TTA and sugar is also reversed, where higher sugar content results in higher TTA values. This is explained by the relatively limited amount of change due to fermentation and less conversion of sugar.

**Figure 6:**
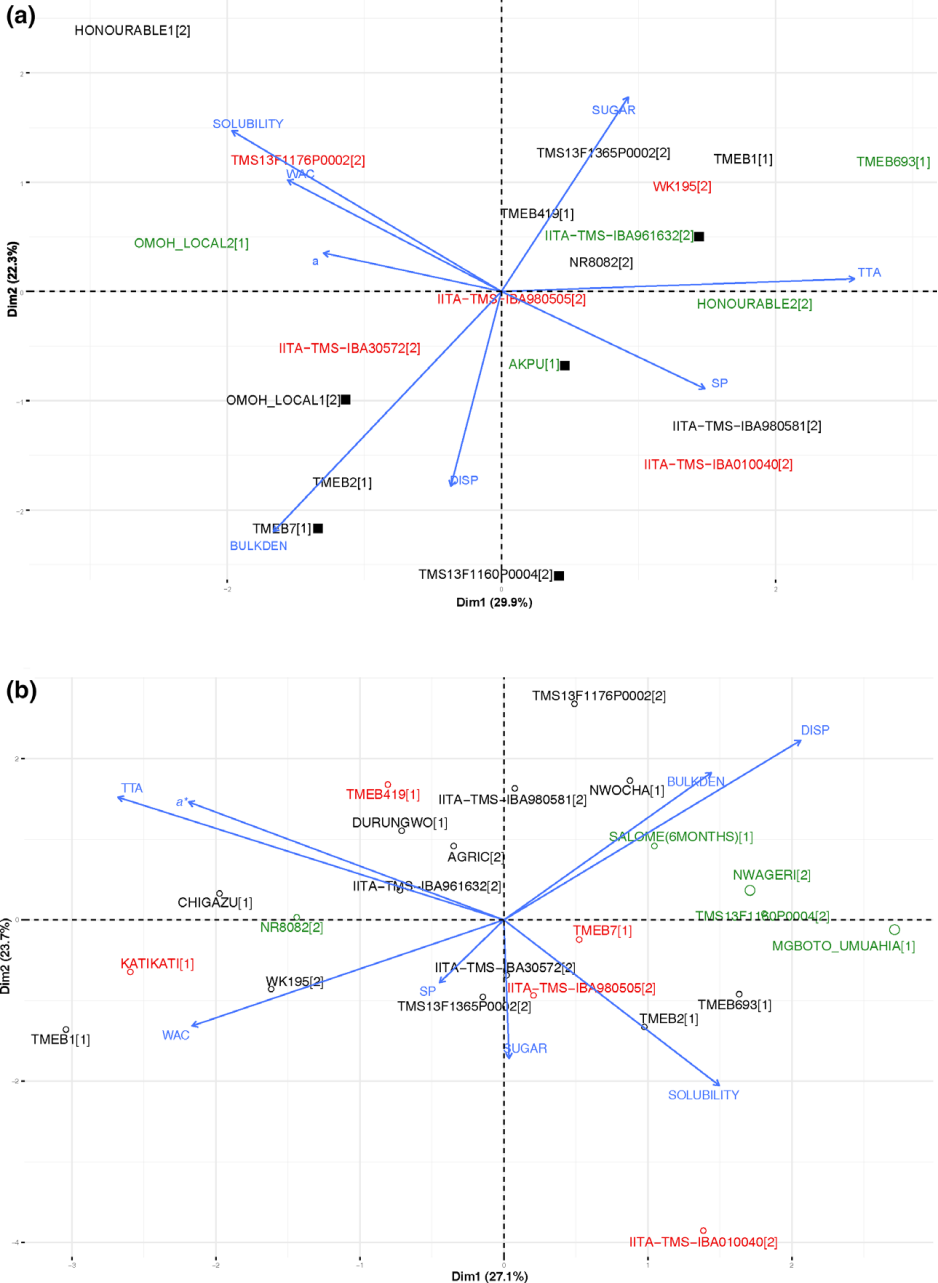
Biplot showing a PCA of the differing laboratory parameters between the best and the worst evaluated varieties measured on the*gari*in Osun (a) and the Imo location (b). The varieties in green are the top five varieties best evaluated for*gari*quality, while those in red are the five lowest ranked. The varieties marked with a black square are the varieties best evaluated for*eba*(Osun only).

Figure 6 shows biplots including laboratory gari parameters that showed significant differences between the best and worst varieties in both Osun and Imo. The best evaluated varieties for eba are marked with a black square for Osun (Fig. 6a). It can be concluded that:

- In Osun (practising thorough fermentation of gari), higher swelling power and titratable acid are determinative for the varieties best for gari and eba, coupled with lower redness (a*), lower solubility (negatively influencing the texture) and lower water absorption capacity. Yield‐related parameters are negatively correlated with food quality.
- In Imo (practising little fermentation of gari), higher bulk density and higher dispersibility are determinative for varieties best for gari, coupled with lower redness (a*), lower titratable acid and lower water absorption capacity. Yield‐related parameters are positively correlated with the food quality.

## Conclusions

In this study, we looked at the suitability of landraces and improved cassava varieties based on root quality criteria used by farmer–processors, the criteria they use to evaluate these varieties for food processing, and links between these criteria and measurable physico‐chemical parameters on roots and food products.

Overall, landraces were more preferred than improved varieties for gari‐making. However, the overall success of the recently improved variety TMS13F1160P0004 shows that it might be possible to develop multipurpose varieties that possess a composite of the requirements by different gari processing cultures, including good gari product qualities. Nonetheless, to obtain *excellent* rather than rather *good* quality within different processing cultures and for both gari and fufu, this study indicated that different variety profiles might be needed. Further research has to establish a clearer picture.

This study showed that the mother trial setup is a good approach to elicit user quality criteria and develop standardised laboratory protocols to process, prepare and evaluate food products, while baby trials can provide important information to weight the impact of breeders’ investments under farmer conditions. The number of baby trials should ideally be much larger and more integrated with breeders’ trials, in order to be able to include more environments and provide more statistical power. Citizen science approaches such as Tricot (Triadic Comparisons of Technologies; van Etten *et al*., 2016) can facilitate such greater farmer involvement in breeding. If good cooperation with farmers is achieved, such methodologies can even be taken up by breeders to improve precision and user suitability of new varieties (Snapp *et al*, 2002) and can be included as an additional cost‐effective strategy within the formal variety release procedures.

We identified the key traits that farmer–processors used to evaluate gari quality: darkening/browning, swelling power, bulk density, solubility, dispersibility and water absorption capacity. Higher water absorption capacity was identified as non‐preferred for gari to make eba. Importantly, we identified that the preferences and importance for some of these parameters is dependent on the processing culture in which either much or little fermentation is practised. Especially a* (degree of redness) – rather than L*(brightness) – related to very important farmer–processors criteria, such as colour and brightness, was identified as the most important criterion and has the advantage of significant heritability. The investigation of the relation between sugar content of the gari and browning, and the possible role of polyphenols and/or phenolic acid in this discoloration, has been identified as an important priority proof of concept.

Efficient breeding will depend on identifying relationships between fresh root quality characteristics and preferred physico‐chemical or functional properties of gari and fufu. Nonetheless, this study illustrated that users were not able to well judge the suitability of a cassava root for food products based only on the evaluation fresh roots. Dry matter content of raw roots, one of the most common quality measures in breeding programmes, was not related to the bulk density of the gari. However, high dry matter was associated with high solubility of gari, a non‐preferred trait there where longer fermentation times are practised. Possible trade‐offs between yield‐related parameters (dry matter and gari yield) and gari quality were identified for fermented gari in Osun state.

Further standardised food science laboratory measurements and protocols of key parameters, especially colour and texture, offer an important platform to test the proof of concept on the relation between physico‐chemical composition of the roots and food product quality. Pasting properties and penetrometer values are two examples of measures that could provide high value in understanding relationships between product quality and physico‐chemical properties This is especially important for identifying rapid screening tools that target both more fermented gari and less fermented gari and eba, as well as fufu.

Such evaluations will have to be incorporated in the breeder evaluations, initially within the later stages of the breeding cycle, but eventually in high‐throughput phenotyping systems at early breeding stages. Thus, it is important to have a verification feedback loop including food processing, which is socially inclusive, cooperative and expertise‐focused, including participatory methodologies.

Finally, incorporating food product quality in cassava breeding objectives would be highly beneficial for the many women in Nigeria. This is in line with an empowerment strategy that builds on local contexts (Adams, 2008), where many vulnerable groups depend upon the food system for their livelihoods.

## Conflict of interest

The authors declare no conflict of interest in this work.

## Author contributions

**Béla Teeken:** Conceptualization‐Lead, Data curation‐Equal, Formal analysis‐Lead, Funding acquisition‐Equal, Investigation‐Equal, Methodology‐Lead, Project administration‐Lead, Resources‐Supporting, Supervision‐Lead, Validation‐Lead, Visualization‐Supporting, Writing‐original draft‐Lead, Writing‐review & editing‐Lead. **Afolabi Agbona:** Conceptualization‐Equal, Data curation‐Lead, Formal analysis‐Lead, Methodology‐Equal, Software‐Lead, Visualization‐Lead, Writing‐original draft‐Equal, Writing‐review & editing‐Equal. **Bello Abolore:** Conceptualization‐Supporting, Data curation‐Equal, Formal analysis‐Supporting, Investigation‐Lead, Methodology‐Supporting, Project administration‐Equal, Resources‐Equal, Validation‐Equal, Writing‐original draft‐Supporting, Writing‐review & editing‐Supporting. **Olamide Olaosebikan:** Conceptualization‐Supporting, Formal analysis‐Equal, Investigation‐Equal, Methodology‐Supporting, Writing‐original draft‐Equal, Writing‐review & editing‐Supporting. **Emmanuel Alamu:** Conceptualization‐Supporting, Formal analysis‐Equal, Investigation‐Supporting, Methodology‐Supporting, Resources‐Equal, Supervision‐Supporting, Validation‐Equal, Writing‐original draft‐Supporting, Writing‐review & editing‐Equal. **Michael Adesokan:** Conceptualization‐Supporting, Data curation‐Equal, Formal analysis‐Supporting, Investigation‐Equal, Methodology‐Supporting, Resources‐Equal, Validation‐Supporting, Writing‐review & editing‐Supporting. **Wasiu Awoyale:** Conceptualization‐Supporting, Formal analysis‐Equal, Methodology‐Supporting, Validation‐Supporting, Writing‐review & editing‐Equal. **Tessy Madu:** Conceptualization‐Equal, Data curation‐Equal, Formal analysis‐Supporting, Investigation‐Lead, Methodology‐Equal, Resources‐Equal, Supervision‐Equal, Validation‐Equal, Writing‐review & editing‐Supporting. **Benjamin Okoye:** Conceptualization‐Supporting, Data curation‐Equal, Formal analysis‐Supporting, Investigation‐Equal, Methodology‐Supporting, Validation‐Supporting. **Ugo Chijioke:** Conceptualization‐Equal, Investigation‐Equal, Methodology‐Supporting, Resources‐Supporting, Validation‐Supporting. **Durodola Owoade:** Data curation‐Supporting, Investigation‐Equal, Resources‐Supporting, Validation‐Supporting. **Maria Okoro:** Data curation‐Supporting, Investigation‐Equal, Methodology‐Supporting, Resources‐Supporting, Validation‐Supporting, Writing‐review & editing‐Supporting. **Alexandre Bouniol:** Data curation‐Supporting, Investigation‐Equal, Methodology‐Supporting, Resources‐Equal, Validation‐Supporting, Writing‐original draft‐Supporting. **Dominique Dufour:** Conceptualization‐Supporting, Formal analysis‐Supporting, Funding acquisition‐Lead, Investigation‐Supporting, Methodology‐Supporting, Supervision‐Equal, Validation‐Supporting, Writing‐original draft‐Supporting, Writing‐review & editing‐Equal. **Clair Hershey:** Visualization‐Supporting, Writing‐review & editing‐Equal. **Ismail Rabbi:** Conceptualization‐Supporting, Formal analysis‐Supporting, Investigation‐Supporting, Methodology‐Supporting, Resources‐Equal, Validation‐Supporting. **Busie Maziya‐Dixon:** Conceptualization‐Supporting, Funding acquisition‐Lead, Investigation‐Supporting, Methodology‐Equal, Supervision‐Supporting, Validation‐Equal, Writing‐original draft‐Supporting, Writing‐review & editing‐Supporting. **Chiedozie Egesi:** Conceptualization‐Equal, Funding acquisition‐Equal, Methodology‐Supporting, Supervision‐Equal, Validation‐Equal, Writing‐review & editing‐Supporting. **Hale Tufan:** Conceptualization‐Equal, Investigation‐Supporting, Methodology‐Equal, Supervision‐Equal, Writing‐original draft‐Supporting, Writing‐review & editing‐Supporting. **Peter Kulakow:** Conceptualization‐Lead, Formal analysis‐Equal, Funding acquisition‐Lead, Methodology‐Equal, Resources‐Equal, Supervision‐Lead, Validation‐Equal, Writing‐original draft‐Supporting, Writing‐review & editing‐Equal.

## Ethical approval

Research teams obtained ethical approval prior to the fieldwork. IITA has the mandate to carry out research in Nigeria including human subjects based on an agreement with the Nigerian government. Participants were informed about the study, they could stop the interview at any point, written consent from sensory panellists and from consumers participating in this study were obtained and the research respected the rules of voluntary participation and anonymity. Food samples were prepared by the participants in this study according to good hygiene and manufacturing practices.

### Peer review

The peer review history for this article is available at https://publons.com/publon/10.1111/ijfs.14862.

## Supporting information


**Table S1.** (a) Overview of the fresh root and product yield related aspects of the mother trial in Osun state. (b) Overview of the fresh root and *gari* yield related aspects of the mother trial in Imo State.
**Table S2.** Performance of the baby trials for dry matter (DM) and fresh yield (FYLD) in Imo and Osun State.
**Table S3.** (a) Bradley‐Terry analysis of the ‘baby trial’ farmer–processors participants pairwise evaluation for fresh roots and gari quality. (b) Bradley‐Terry analysis of the ‘baby trial’ farmer–processors participants pairwise evaluation for fresh roots and gari quality.
**Table S4.** (a) Functional properties of gari produced from improved and landrace cassava varieties in Osun State. (b) Functional properties of gari produced from improved and landrace cassava varieties in Imo State.
**Table S5.** (a) Physical colour and chemical properties of gari produced from improved and landrace cassava varieties in Osun State. (b) Physical colour and chemical properties of gari produced from improved and landrace cassava varieties in Imo State.
**Table S6.** (a) Pearson correlation of the functional and chemical properties and the physical colour of gari compared with the root dry matter and gari yield from different varieties in Osun State. (b) Pearson correlation of the functional and chemical properties and the physical colour of gari compared with the root dry matter and gari yield from different varieties in Imo State.

## Data Availability

The data that support the findings of this study are available from the corresponding author upon reasonable request.
