# Supplementary material for: Understanding cassava varietal preferences through pairwise ranking of gari‐eba and fufu prepared by local farmer–processors
Source: Int J Food Sci Technol. 2020 Nov 20;56(3):1258–77. doi: 10.1111/ijfs.14862 (PMC7984147; doi:10.1111/ijfs.14862)
Supplement: Supplementary file 1 — Table S1. (a) Overview of the fresh root and product yield related aspects of the mother trial in Osun state. (b) Overview of the fresh root and gari yield related aspects of the mother trial in Imo State. Table S2. Performance of the baby trials for dry matter (DM) and fresh yield (FYLD) in Imo and Osun State. Table S3. (a) Bradley‐Terry analysis of the ‘baby trial’ farmer–processors participants pairwise evaluation for fresh roots and gari quality. (b) Bradley‐Terry analysis of the ‘baby trial’ farmer–processors participants pairwise evaluation for fresh roots and gari quality. Table S4. (a) Functional properties of gari produced from improved and landrace cassava varieties in Osun State. (b) Functional properties of gari produced from improved and landrace cassava varieties in Imo State. Table S5. (a) Physical colour and chemical properties of gari produced from improved and landrace cassava varieties in Osun State. (b) Physical colour and chemical properties of gari produced from improved and landrace cassava varieties in Imo State. Table S6. (a) Pearson correlation of the functional and chemical properties and the physical colour of gari compared with the root dry matter and gari yield from different varieties in Osun State. (b) Pearson correlation of the functional and chemical properties and the physical colour of gari compared with the root dry matter and gari yield from different varieties in Imo State. [file IJFS-56-1258-s001.docx]

**Supplementary Tables**

**Table S1a.** Overview of the fresh root and product yield related aspects of the mother trial in Osun state

| Variety | Dry matter | Dry root Yield | Fresh root yield | Gari yield (Kg) | Gari yield (%) | 'Pounded' Fufu Yield | 'Turned' Fufu Yield |
| --- | --- | --- | --- | --- | --- | --- | --- |
| **Improved** |  |  |  |  |  |  |  |
| HONOURABLE1 | 34.8 | 10.9 | 31.2 | 7.21 | 36.1 | 4.59 | 4.47 |
| HONOURABLE2 | 35.7 | 13.2 | 36.9 | 5.91 | 29.6 | 5.90 | 5.03 |
| IITA-TMS-IBA010040 | 32.8 | 13.2 | 39.8 | 6.99 | 34.9 | 5.80 | 5.63 |
| IITA-TMS-IBA30572 | 37.8 | 9.5 | 25.20 | 7.18 | 35.9 | 6.98 | 5.94 |
| IITA-TMS-IBA961632 | 37.0 | 14.0 | 37.9 | 6.69 | 33.4 | 5.18 | 5.01 |
| IITA-TMS-IBA980505 | 32.9 | 13.8 | 42.0 | 7.77 | 38.9 | 5.01 | 4.32 |
| IITA-TMS-IBA980581 | 33.9 | 10.3 | 30.4 | 7.38 | 36.9 | 6.26 | 5.17 |
| NR8082 | 33.1 | 10.1 | 30.6 | 6.73 | 33.6 | 4.24 | 3.82 |
| OMOH_LOCAL1 | 35.4 | 7.6 | 21.5 | 6.69 | 33.4 | 6.52 | 5.56 |
| TMS13F1160P0004 | 38.9 | 11.6 | 29.8 | 7.28 | 36.4 | 5.15 | 5.21 |
| TMS13F1176P0002 | 37.5 | 8.0 | 21.6 | 6.80 | 34.0 | 6.77 | 5.57 |
| TMS13F1365P0002 | 36.6 | 11.6 | 32.6 | 6.82 | 34.1 | 5.93 | 5.32 |
| WK195 | 33.9 | 11.6 | 34.7 | 6.51 | 32.6 | 2.42 | 2.21 |
| **Landraces** |  |  |  |  |  |  |  |
| AKPU | 34.7 | 9.8 | 28.2 | 6.50 | 32.5 | 6.37 | 5.21 |
| OMOH_LOCAL2 | 35.9 | 8.7 | 24.3 | 7.53 | 37.6 | 5.80 | 4.87 |
| TMEB1 | 35.5 | 8.2 | 23.1 | 6.49 | 32.5 | 6.16 | 5.43 |
| TMEB2 | 37.7 | 8.5 | 22.40 | 7.50 | 37.5 | 6.22 | 5.33 |
| TMEB419 | 38.3 | 9.9 | 25.7 | 7.16 | 35.8 | 7.13 | 6.28 |
| TMEB693 | 35.4 | 7.8 | 22.0 | 7.17 | 35.9 | 6.71 | 5.88 |
| TMEB7 | 35.4 | 7.5 | 21.6 | 7.69 | 38.5 | 5.14 | 4.86 |
| Mean | 35.7 | 10.3 | 29.1 | 7.00 | 35.0 | 5.71 | 5.06 |
| Standard Error | 0.99 | 3.16 | 8.90 | 0.47 | 2.35 | 1.89 | 1.67 |
| Min | 32.78 | 7.51 | 21.46 | 5.91 | 29.56 | 2.42 | 2.21 |
| Max | 38.89 | 13.97 | 42.03 | 7.77 | 38.87 | 7.13 | 6.28 |
| Genotypic variance | 2.41 | 0.00 | 3.64 | 0.06 | 0.29 | 0.69 | 0.34 |
| Genotype x Year variance | 0.48 | 6.97 | 54.09 | 0.30 | 1.48 | 1.06 | 0.85 |
| Residual variance | 3.81 | 9.44 | 76.49 | 0.10 | 0.48 | 0.05 | 0.08 |
| Broad sense heritability | 0.73 | 0.00 | 0.08 | 0.26 | 0.26 | 0.56 | 0.43 |
| Genotypic CV | 4.35 | 0.00 | 6.57 | 3.47 | 3.47 | 14.55 | 11.47 |
| Phenotypic CV | 5.38 | 17.24 | 18.57 | 4.31 | 4.31 | 14.72 | 11.91 |
| Least Significant Difference | 2.77 | 6.99 | 18.67 | 1.20 | 6.00 | 2.17 | 1.96 |
| CV | 3.71 | 32.46 | 30.69 | 8.18 | 8.18 | 18.16 | 18.55 |

N.B. DM=Root Dry Matter (%), DYLD= Dry root yield (ton/ha), FYLD=Fresh Root Yield (ton/ha), GARI YLD= Gari Yield (kg per 20kg of peeled roots), POUND FUFU YLD=Pounded fufu yield (kg per 20kg peeled roots), TURN FUFU YLD= Turned fufu yield (kg per 20kg peeled roots).

**Table S1b.** Overview of the fresh root and *gari* yield related aspects of the mother trial in Imo State

| Variety | Dry matter | Dry root Yield | Fresh root yield | Gari yield (Kg) | Gari yield (%) |
| --- | --- | --- | --- | --- | --- |
| **Improved** |  |  |  |  |  |
| AGRIC | 29.8 | 4.9 | 16.1 | 7.32 | 36.6 |
| IITA-TMS-IBA010040 | 26.3 | 4.5 | 17.3 | 5.09 | 25.5 |
| IITA-TMS-IBA30572 | 31.4 | 4.1 | 13.0 | 5.98 | 29.9 |
| IITA-TMS-IBA961632 | 32.5 | 5.4 | 16.6 | 6.76 | 33.8 |
| IITA-TMS-IBA980505 | 31.9 | 7.2 | 22.4 | 5.87 | 29.4 |
| IITA-TMS-IBA980581 | 30.6 | 7.3 | 23.6 | 6.78 | 33.9 |
| NR8082 | 29.2 | 5.7 | 19.9 | 5.86 | 29.3 |
| NWAGERI | 31.2 | 5.5 | 17.4 | 6.95 | 34.8 |
| TMS13F1160P0004 | 35.6 | 6.2 | 17.2 | 8.41 | 42.1 |
| TMS13F1176P0002 | 34.3 | 5.5 | 16.2 | 7.90 | 39.5 |
| TMS13F1365P0002 | 34.3 | 7.3 | 21.1 | 6.86 | 34.3 |
| WK195 | 31.7 | 6.5 | 20.8 | 5.43 | 27.2 |
| **Landraces** |  |  |  |  |  |
| KATIKATI | 26.6 | 4.1 | 15.0 | 5.07 | 25.3 |
| MGBOTO_UMUAHIA | 34.3 | 8.1 | 23.8 | 8.19 | 40.9 |
| CHIGAZU | 31.2 | 3.6 | 11.1 | 7.61 | 38.1 |
| DURUNGWO | 31.8 | 5.6 | 17.4 | 7.74 | 38.7 |
| NWOCHA | 33.4 | 7.5 | 22.6 | 7.60 | 38.0 |
| SALOME(6MONTHS) | 29.5 | 6.9 | 23.0 | 6.95 | 34.8 |
| TMEB1 | 28.7 | 3.2 | 10.9 | 5.66 | 28.3 |
| TMEB2 | 31.6 | 2.9 | 9.1 | 5.84 | 29.2 |
| TMEB419 | 33.4 | 3.2 | 9.6 | 7.15 | 35.7 |
| TMEB693 | 31.2 | 4.6 | 13.6 | 7.17 | 35.9 |
| TMEB7 | 30.6 | 4.1 | 13.2 | 6.92 | 34.6 |
| Mean | 31.3 | 5.4 | 17.0 | 6.74 | 33.7 |
| Standard Error | 1.10 | 1.48 | 4.54 | 0.90 | 4.51 |
| Min | 26.33 | 2.89 | 9.08 | 5.07 | 25.33 |
| Max | 35.59 | 8.05 | 23.81 | 8.41 | 42.06 |
| Genotypic variance | 4.30 | 0.69 | 5.73 | 0.64 | 3.22 |
| Genotype x Year variance | 0.86 | 1.51 | 10.75 | 0.59 | 2.95 |
| Residual variance | 4.46 | 5.79 | 60.10 | 0.06 | 0.29 |
| Broad sense heritability | 0.79 | 0.29 | 0.27 | 0.68 | 0.68 |
| Genotypic CV | 6.61 | 15.45 | 14.09 | 11.89 | 11.89 |
| Phenotypic CV | 7.67 | 30.12 | 29.87 | 12.07 | 12.07 |
| Least Significant Difference | 3.17 | 3.85 | 11.51 | 1.62 | 8.10 |
| CV | 4.88 | 34.55 | 32.65 | 11.58 | 11.58 |

N.B. DM=Root Dry Matter (%), DYLD= Dry root yield (ton/ha), FYLD=Fresh Root Yield (ton/ha), GARI YLD= Gari Yield (kg per 20kg of peeled roots).

**Table S2.** Performance of the baby trials for dry matter (DM) and fresh yield (FYLD) in Imo and Osun State

|  | Imo | |  | **Osun** | |
| --- | --- | --- | --- | --- | --- |
| Variety | Dry matter (%) | Fresh yield (ton/ha) |  | Dry matter (%) | Fresh yield (ton/ha) |
| **Improved** |  |  |  |  |  |
| IITA-TMS-IBA010040 | 28.5 | 10.5 |  | 34.5 | 32.5 |
| IITA-TMS-IBA30572 | 34.1 | 3.5 |  | 32.0 | 10.8 |
| IITA-TMS-IBA961632 | 39.1 | 20.7 |  | 38.0 | 16.9 |
| IITA-TMS-IBA980505 | 37.6 | 21.1 |  | 35.5 | 36.4 |
| IITA-TMS-IBA980581 | 31.0 | 13.3 |  | 33.6 | 18.8 |
| NR8082 | 30.8 | 5.9 |  | 33.3 | 10.0 |
| TMS13F1160P0004 | 40.6 | 8.8 |  | 34.2 | 23.8 |
| TMS13F1176P0002 | 36.8 | 10.8 |  | 38.4 | 18.8 |
| TMS13F1365P0002 | 33.9 | 19.3 |  | 35.9 | 25.0 |
| WK195 | 27.0 | 6.2 |  | 30.4 | 16.8 |
| **Landraces** |  |  |  |  |  |
| TMEB1 | 31.0 | 16.1 |  | 37.4 | 15.4 |
| TMEB2 | 31.6 | 5.6 |  | 31.9 | 14.8 |
| TMEB419 | 31.2 | 9.2 |  | 36.9 | 18.3 |
| TMEB693 | 34.1 | 13.0 |  | 34.3 | 17.0 |
| TMEB7 | 35.4 | 16.5 |  | 37.3 | 13.4 |
| ***Improved local Osun*** |  |  |  |  |  |
| *FULANI* |  |  |  | 39.5 | 6.5 |
| *IITA* |  |  |  | 34.3 | 14.6 |
| *AGRIC* |  |  |  | 32.2 | 38.3 |
| *IITA-YELLOW* |  |  |  | 21.5 | 8.0 |
| ***Landraces local Osun*** |  |  |  |  |  |
| *AWOLOWO* |  |  |  | 36.3 | 11.0 |
| *WHITE CASSAVA* |  |  |  | 35.5 | 5.8 |
| *OKO-IYAWO* |  |  |  | 35.5 | 19.6 |
| *ATU* |  |  |  | 35.2 | 26.3 |
| *AKPU* |  |  |  | 34.5 | 8.1 |
| *NIKAN-PUPA* |  |  |  | 33.3 | 20.0 |
| ***Improved local Imo*** |  |  |  |  |  |
| *AGRIC* | 35.2 | 13.3 |  |  |  |
| *NWAGERI* | 33.9 | 16.0 |  |  |  |
| ***Landraces local Imo*** |  |  |  |  |  |
| *MGBOTO_UMUAHIA* | 41.9 | 6.5 |  |  |  |
| *CHIGAZU* | 34.8 | 15.8 |  |  |  |
| *ADA_NWANKWO* | 33.5 | 7.9 |  |  |  |
| *SALOME* | 30.7 | 19.7 |  |  |  |
| Mean | 33.9 | 12.4 |  | 34.5 | 17.9 |
| Standard Error | 2.38 | 4.19 |  | 1.63 | 5.28 |
| Min | 27.02 | 3.50 |  | 21.53 | 5.83 |
| Max | 41.89 | 21.08 |  | 39.46 | 38.33 |
| Genotypic variance | 8.48 | 13.47 |  | 9.46 | 46.64 |
| Residual variance | 11.84 | 32.26 |  | 5.05 | 58.07 |
| Broad sense heritabilty | 0.59 | 0.46 |  | 0.79 | 0.62 |
| Genotypic CV | 8.58 | 29.68 |  | 8.93 | 38.20 |
| Phenotypic CV | 11.18 | 44.00 |  | 10.05 | 48.66 |
| Least Significant Difference | 7.00 | 11.85 |  | 4.80 | 15.38 |
| CV | 9.92 | 45.93 |  | 6.73 | 41.78 |

N.B. varieties in italics are the varieties each of the baby trial participants have added to the trial as their personal favourite

**Table S3a.** Bradley-Terry analysis of the ‘baby trial’ farmer-processors participants pairwise evaluation for fresh roots and gari quality. Values indicate total of pairwise ranks of fresh roots ‘for gari’, gari and eba based on one-year data from baby trials in Osun state. Varieties with higher estimates are preferred more often in paired comparisons than those with lower estimates. Results from the best five and worst five varieties are highlighted in green and red, respectively, for fresh roots ‘for gari’, gari and eba. The groups represent the landraces (1) and Improved varieties (2).

| **Group/Variety** | **Group** | **Fresh roots for gari** | **Gari** | **Eba** |
| --- | --- | --- | --- | --- |
|  |  |  |  |  |
| **Local** |  |  |  |  |
| AGRIC | 2 | 1.46 | 2.00* | -8.27 |
| ATU | 1 | -0.57 | 0.87 | -10.82 |
| Awolowo | NA | -0.19 | 17.20 | -8.22 |
| Fulani | 2 | 15.91 | 15.67 | -10.95 |
| IITA | 2 | -1.46 | 1.24 | -11.21 |
| LC | 1 | -0.12 | 2.62** | -10.16 |
| NIKAN PUPA | 1 | 14.85 | 16.44 | -0.62 |
| OKO-IYAWO | 1 | -0.02 | 1.63 | -9.83 |
| WHITE CASSAVA | NA | -15.14 | 17.16 | -10.27 |
|  |  |  |  |  |
| **Common landraces** |  |  |  |  |
| TMEB1 | 1 | -0.90 | 0.97 | -10.90 |
| TMEB2 | 1 | -1.37 | 1.65* | -10.88 |
| TMEB419 | 1 | 0.67 | 1.75* | -10.97 |
| TMEB693 | 1 | -0.47 | 2.04** | -10.33 |
| TMEB7 | 1 | 1.02 | -2.64* | -11.53 |
|  |  |  |  |  |
| **IITA/NRCRI Improved** |  |  |  |  |
|  |  |  |  |  |
| IITA-TMS-IBA010040 | 2 | 1.01 | 0.23 | -9.96 |
| IITA-TMS-IBA30572 | 2 | -1.38 | 2.22** | -7.50 |
| IITA-TMS-IBA961632 | 2 | 0.67 | 2.33** | -9.56 |
| IITA-TMS-IBA980505 | 2 | 0.70 | 1.69* | -11.53 |
| IITA-TMS-IBA980581 | 2 | 1.39 | 0.58 | -9.69 |
| NR8082 | 2 | -0.52 | 1.61* | -10.20 |
| TMS13F1160P0004 | 2 | 0.20 | 2.75*** | -7.23 |
| TMS13F1176P0002 | 2 | -1.07 | -1.18 | -11.50 |
| TMS13F1365P0002 | 2 | 1.65 | 1.98* | -10.94 |
| WK195 | 2 | 0.63 | 0.00 | -12.63 |

Significance levels indicate the probability that the estimate is significantly different from 0: * = *p* <0.10; ** = *p* < 0.05, ***= *p* <0.010, ****=*p* <0.001. NA= not assessed (not genetically analysed)

**Table S3b.** Bradley-Terry analysis of the ‘baby trial’ farmer-processors participants pairwise evaluation for fresh roots and gari quality. Values indicate total of pairwise ranks of fresh roots ‘for gari’ and gari based on one-year data from baby trials in Imo state. Varieties with higher estimates are preferred more often in paired comparisons than those with lower estimates. Results from the best five and worst five varieties are highlighted in green and red, respectively, for fresh roots ‘for gari’ and gari. The groups represent the landraces (1) and Improved varieties (2)

| **Group/Variety** | **group** | **Fresh roots for gari** | **Gari** |
| --- | --- | --- | --- |
|  |  |  |  |
| **Local** |  |  |  |
|  |  |  |  |
| ADA_NWANKWO | NA | -2.53** | -0.99 |
| AGRIC | 2 | 13.40 | -1.07 |
| CHIGAZU | 1 | -1.48*** | 0.04 |
| MGBOTO_UMUAHIA | 1 | 13.42 | 15.27 |
| NWAGERI | 2 | -2.10**** | 0.14 |
| SALOME | 1 | -2.57** | -2.13* |
|  |  |  |  |
| **Common landraces** |  |  |  |
|  |  |  |  |
| TMEB1 | 1 | -2.79**** | -1.15** |
| TMEB2 | 1 | -3.65**** | -0.01 |
| TMEB419 | 1 | -1.79**** | 0.25 |
| TMEB693 | 1 | -1.05* | 0.21 |
| TMEB7 | 1 | -1.86**** | 0.31 |
|  |  |  |  |
| **IITA/NRCRI Improved** |  |  |  |
|  |  |  |  |
| IITA-TMS-IBA010040 | 2 | -1.35** | -0.69 |
| IITA-TMS-IBA30572 | 2 | -2.39**** | 0.42 |
| IITA-TMS-IBA961632 | 2 | -1.47** | -0.20 |
| IITA-TMS-IBA980505 | 2 | -1.62*** | -0.29 |
| IITA-TMS-IBA980581 | 2 | -1.44** | 0.22 |
| TMS13F1160P0004 | 2 | -1.46*** | 1.39*** |
| TMS13F1176P0002 | 2 | -2.58**** | -0.07 |
| TMS13F1365P0002 | 2 | -1.11** | 0.13 |
| WK195 | 2 | 0.00 | 0.00 |
| NR8082 | 2 | -2.43**** | -1.17** |

Significance levels indicate the probability that the estimate is significantly different from 0: * = *p* <0.10; ** = *p* < 0.05, ***= *p* <0.010, ****=*p* <0.001. NA= not assessed (not genetically analysed)

**Table S4a**. Functional properties of gari produced from improved and landrace cassava varieties in Osun State

| Varieties | N | Water absorption capacity (%) | Swelling power (%) | Solubility index (%) | Dispersibility (%) | Bulk density (%) |
| --- | --- | --- | --- | --- | --- | --- |
| **Improved** |  |  |  |  |  |  |
| HONOURABLE1 | 12 | 515.16±41.22a | 5.77±0.30f | 7.97±0.69a | 39.50±3.71a-c | 55.79±3.40a-d |
| HONOURABLE2 | 12 | 469.07±24.61a-c | 7.18±1.14bc | 6.41±0.49d-f | 40.08±1.08a-c | 54.87±3.72a-e |
| IITA-TMS-IBA010040 | 12 | 460.59±28.28a-c | 6.98±1.57b-d | 6.27±0.78ef | 42.50±5.18ab | 58.04±3.03a |
| IITA-TMS-IBA30572 | 12 | 482.19±24.22a-c | 6.34±1.17c-f | 7.18±0.83a-d | 43.75±4.20a | 55.69±2.75a-d |
| IITA-TMS-IBA961632 | 12 | 451.90±73.84c | 6.43±0.35b-f | 7.11±0.52a-e | 39.50±2.35a-c | 53.50±3.33b-e |
| IITA-TMS-IBA980505 | 12 | 474.06±40.48a-c | 6.22±0.57d-f | 6.60±0.31c-f | 39.00±2.35a-c | 56.17±2.13a-d |
| IITA-TMS-IBA980581 | 12 | 478.85±26.32a-c | 8.76±0.62a | 6.54±1.08c-f | 41.92±3.75a-c | 55.93±2.15a-d |
| NR8082 | 12 | 450.99±38.04c | 6.22±0.56d-f | 6.82±0.54b-f | 42.17±2.69a-c | 54.41±4.62a-e |
| OMOH_LOCAL1 | 12 | 512.72±48.08ab | 6.51±1.00b-f | 6.84±0.69b-f | 42.08±4.89a-c | 57.26±4.09a-c |
| TMS13F1160P0004 | 12 | 456.24±32.83bc | 5.90±0.66ef | 6.22±0.24f | 43.08±4.81ab | 57.74±3.34ab |
| TMS13F1176P0002 | 12 | 501.85±43.82a-c | 6.76±1.53b-e | 7.06±1.20b-f | 41.33±3.26a-c | 55.63±4.07a-d |
| TMS13F1365P0002 | 12 | 496.88±67.48a-c | 6.63±0.39b-f | 6.59±0.58c-f | 37.42±3.53c | 53.07±7.95c-e |
| WK195 | 12 | 465.06±54.29a-c | 6.41±0.63b-f | 7.02±0.57b-f | 41.33±3.80a-c | 52.49±3.34de |
| **Landraces** |  |  |  |  |  |  |
| OMOH_LOCAL2 | 12 | 503.37±29.06a-c | 6.00±0.50ef | 7.34±0.89a-c | 38.67±1.87bc | 56.24±2.62a-d |
| AKPU | 12 | 480.50±35.75a-c | 7.30±1.60b | 6.36±0.50d-f | 39.33±3.96a-c | 56.95±1.51a-c |
| TMEB1 | 12 | 499.86±22.96a-c | 7.13±0.51b-d | 6.44±0.80d-f | 38.58±2.27bc | 53.36±2.84c-e |
| TMEB2 | 12 | 470.66±63.25a-c | 7.10±1.49b-d | 6.83±0.95b-f | 42.00±3.54a-c | 58.60±2.54a |
| TMEB419 | 12 | 445.89±59.30c | 6.20±0.25d-f | 7.54±0.66ab | 42.00±3.30a-c | 53.55±3.74b-e |
| TMEB693 | 12 | 462.65±41.31a-c | 7.33±1.14b | 6.29±0.67ef | 39.25±3.96a-c | 50.75±6.15e |
| TMEB7 | 12 | 470.66±70.61a-c | 7.10±1.49b-d | 6.83±0.95b-f | 42.00±3.54a-c | 57.74±2.54a |
| Improved varieties mean |  | 478.12 | 6.62 | 6.82 | 41.05 | 55.43 |
| Landrace varieties mean |  | 476.23 | 6.88 | 6.80 | 40.26 | 55.44 |
| Range |  | 336.32-594.74 | 5.11-9.90 | 5.36-16.64 | 25.00-49.00 | 44.34-65.30 |
| Year |  | NS | NS | NS | NS | NS |
| Varieties |  | *** | *** | *** | *** | *** |
| Year x Varieties |  | *** | *** | *** | *** | *** |

**Table S4b**. Functional properties of gari produced from improved and landrace cassava varieties in Imo State

| Varieties | N | Water absorption capacity (%) | Swelling power  (%) | Solubility index (%) | Dispersibility (%) | Bulk density  (%) |
| --- | --- | --- | --- | --- | --- | --- |
| **Improved** |  |  |  |  |  |  |
| AGRIC | 12 | 501.71±36.86a-d | 8.39±1.14c-h | 9.74±1.02c-g | 32.50±4.17a-c | 62.71±1.79a-c |
| IITA-TMS-IBA010040 | 12 | 488.00±46.34b-f | 8.62±0.83c-g | 13.03±1.56a | 23.17±7.54e-g | 55.45±3.77i-k |
| IITA-TMS-IBA30572 | 12 | 459.68±39.81c-f | 8.49±1.23c-g | 10.78±1.40a-e | 20.83±4.37fg | 59.24±1.79c-h |
| IITA-TMS-IBA961632 | 12 | 465.38±15.06b-f | 9.86±0.92a-c | 10.07±0.49c-g | 27.67±3.45c-e | 61.88±1.09a-e |
| IITA-TMS-IBA980505 | 12 | 508.26±26.30a-d | 8.32±0.32c-h | 11.58±0.24a-d | 29.83±4.49b-d | 58.90±1.10e-i |
| IITA-TMS-IBA980581 | 12 | 457.78±33.16c-f | 6.84±0.89hi | 9.33±0.99d-g | 32.08±3.60a-c | 60.48±1.55 |
| NR8082 | 12 | 523.78±20.48ab | 9.66±0.38a-d | 8.32±0.21fg | 23.67±1.56d-g | 62.43±1.83a-d |
| NWAGERI | 12 | 434.32±80.70e-h | 7.25±0.93f-i | 11.01±0.50a-e | 29.92±4.83b-d | 61.49±2.00a-g |
| TMS13F1160P0004 | 12 | 378.52±48.40h | 7.22±0.50f-i | 11.91±0.68a-c | 30.00±2.83b-d | 58.71±0.43e-j |
| TMS13F1176P0002 | 12 | 448.33±41.63d-g | 7.83±1.06e-i | 8.98±0.57e-g | 35.42±3.32ab | 63.97±2.15a |
| TMS13F1365P0002 | 12 | 429.99±68.78f-h | 10.39±1.62ab | 10.25±2.18b-g | 28.17±3.74c-e | 53.19±1.73k |
| WK195 | 12 | 517.80±33.26a-c | 8.64±1.86c-g | 10.09±1.78c-g | 24.50±2.39d-g | 56.60±1.52g-k |
| **Landraces** |  |  |  |  |  |  |
| CHIGAZU | 12 | 471.67±8.88b-f | 10.91±0.44a | 8.14±0.24g | 26.83±2.12c-f | 55.44±2.21i-k |
| DURUNGWO | 12 | 475.49±23.25b-f | 7.69±0.81e-i | 10.53±0.41b-f | 29.50±4.93b-e | 59.77±3.17b-g |
| KATIKATI | 12 | 495.53±24.68a-e | 8.95±1.71b-e | 10.47±0.92b-g | 19.33±8.13g | 55.91±3.32h-k |
| MGBOTO_UMUAHIA | 12 | 467.71±33.10b-f | 8.67±0.82c-f | 12.57±2.43ab | 34.83±3.69ab | 62.80±1.67ab |
| NWOCHA | 12 | 392.32±16.25gh | 9.82±1.53a-c | 9.30±0.85d-g | 38.17±1.53a | 58.31±1.84f-j |
| SALOME(6MONTHS) | 12 | 431.81±62.72e-h | 8.15±0.80d-h | 10.29±1.60b-g | 31.92±1.56a-c | 60.64±4.48a-g |
| TMEB1 | 12 | 553.71±12.43a | 7.05±0.66g-i | 11.61±1.45a-d | 19.67±5.93g | 55.27±1.89jk |
| TMEB2 | 12 | 470.77±39.00b-f | 8.89±0.54b-e | 10.04±2.20c-g | 26.67±3.60c-f | 60.32±0.95 |
| TMEB419 | 12 | 461.78±48.28b-f | 6.25±0.40i | 10.19±0.80b-g | 33.00±3.67a-c | 59.22±0.53d-h |
| TMEB693 | 12 | 456.48±59.69c-f | 8.81±0.98b-f | 11.20±3.86a-e | 32.50±2.68a-c | 60.06±3.09b-g |
| TMEB7 | 12 | 461.78±48.28b-f | 8.46±1.44c-g | 10.36±2.23b-g | 31.17±5.70bc | 61.49±2.51a-g |
| Improved varieties mean | | 467.8 | 8.46 | 10.42 | 28.15 | 59.59 |
| Landrace varieties mean | | 467.48 | 8.51 | 10.43 | 29.42 | 59.02 |
| Range |  | 326.09-571.43 | 5.74-12.48 | 7.87-16.64 | 10.00-41.00 | 49.78-67.22 |
| Year |  | NS | NS | NS | NS | NS |
| Varieties |  | *** | *** | *** | *** | *** |
| Year x Varieties |  | NS | NS | NS | NS | NS |

**Table S5a**. Physical colour and chemical properties of gari produced from improved and landrace cassava varieties in Osun State

| Varieties | N | L* | a* | b* | Sugar content (%) | Starch content (%) | Total titratable acidity (g/100 ml) |
| --- | --- | --- | --- | --- | --- | --- | --- |
| **Improved** |  |  |  |  |  |  |  |
| HONOURABLE1 | 12 | 84.75±4.67ab | 2.41±1.39a-c | 22.19±10.35b-g | 3.31±0.28a-c | 87.56±2.48a | 0.49±0.05j |
| HONOURABLE2 | 12 | 82.18±5.83a-c | 1.80±0.47cd | 24.62±7.60a-e | 3.17±0.52a-c | 83.06±4.21cd | 0.65±0.10b-f |
| IITA-TMS-IBA010040 | 12 | 82.57±3.83a-c | 1.82±0.70cd | 25.11±7.73a-e | 3.22±0.68a-d | 82.52±3.37cd | 0.67±0.10a-c |
| IITA-TMS-IBA30572 | 12 | 81.77±5.86a-c | 2.57±0.95a-c | 25.24±10.97a-d | 3.04±0.28b-e | 84.57±4.83a-d | 0.60±0.08b-h |
| IITA-TMS-IBA961632 | 12 | 82.63±3.49a-c | 2.33±0.87a-d | 22.28±8.34b-g | 2.96±0.76b-e | 83.34±3.06cd | 0.62±0.10b-h |
| IITA-TMS-IBA980505 | 12 | 86.31±1.90a | 1.84±0.86cd | 19.77±5.69g | 3.02±0.64b-e | 81.53±3.81d | 0.58±0.07f-i |
| IITA-TMS-IBA980581 | 12 | 81.69±3.96a-c | 2.20±0.91b-d | 23.64±6.49a-f | 3.09±0.47b-e | 82.10±3.22cd | 0.66±0.12a-e |
| NR8082 | 12 | 86.36±3.18a | 1.85±1.02cd | 23.56±12.38a-f | 3.35±0.35ab | 83.36±4.43cd | 0.59±0.05d-h |
| OMOH_LOCAL1 | 12 | 78.16±6.08c | 2.40±1.08a-c | 21.81±6.69d-g | 2.84±0.45de | 83.56±5.21b-d | 0.59±0.08c-h |
| TMS13F1160P0004 | 12 | 86.67±5.02a | 1.48±0.68d | 21.40±6.44e-g | 2.83±0.61de | 82.06±4.09cd | 0.57±0.04g-j |
| TMS13F1176P0002 | 12 | 81.75±6.63a-c | 3.16±1.22a | 21.57±9.44d-g | 3.52±0.49a | 84.64±4.67a-d | 0.55±0.09h-j |
| TMS13F1365P0002 | 12 | 85.59±2.44a | 2.10±0.86b-d | 26.27±6.94a | 3.04±0.62b-e | 82.40±5.69cd | 0.64±0.09b-g |
| WK195 | 12 | 86.48±3.23a | 1.87±1.04cd | 21.40±10.10e-g | 3.27±0.73a-c | 82.38±2.68cd | 0.67±0.09ab |
| **Landraces** |  |  |  |  |  |  |  |
| OMOH_LOCAL2 | 12 | 86.55±2.37a | 1.90±1.12cd | 22.73±11.54a-g | 2.82±0.21de | 86.77±8.53ab | 0.51±0.03ij |
| AKPU | 12 | 83.79±8.34a-c | 2.36±1.82a-c | 20.41±7.18fg | 3.00±0.37b-e | 82.23±4.32cd | 0.58±0.07e-i |
| TMEB1 | 12 | 79.47±5.98bc | 2.64±1.27a-c | 22.42±5.89b-g | 3.12±0.31a-e | 83.35±6.26cd | 0.73±0.06a |
| TMEB2 | 12 | 84.31±3.66ab | 2.50±0.97a-c | 25.95±8.27ab | 2.93±0.36c-e | 82.47±3.47cd | 0.56±0.06h-j |
| TMEB419 | 12 | 83.18±1.16a-c | 1.92±0.85cd | 22.00±6.03c-g | 3.25±0.32a-c | 85.16±4.04a-c | 0.62±0.10b-h |
| TMEB693 | 12 | 82.41±6.79a-c | 1.90±0.58cd | 25.72±8.39a-c | 3.34±0.61ab | 83.36±2.97cd | 0.66±0.06a-d |
| TMEB7 | 12 | 81.63±3.38a-c | 2.84±0.61ab | 23.15±7.64a-g | 2.72±0.47e | 83.05±3.96cd | 0.56±0.06h-j |
| Improved mean |  | 83.61 | 2.14 | 22.99 | 3.13 | 83.31 | 0.61 |
| Landrace mean |  | 83.05 | 2.30 | 23.20 | 3.03 | 83.77 | 0.6 |
| Range |  | 67.84-93.33 | 1.48 – 3.16 | 11.04-38.98 | 2.11-7.58 | 59.76-96.73 | 0.05-0.82 |
| Year |  | ** | *** | *** | *** | *** | NS |
| Varieties |  | *** | *** | *** | *** | *** | *** |
| Year x Varieties |  | *** | *** | *** | *** | *** | *** |

**Table S5b**. Physical colour and chemical properties of gari produced from improved and landrace cassava varieties in Imo State

| Varieties | N | L* | a* | b* | Sugar content (%) | Starch content (%) | Total titratable acidity (g/100 ml) |
| --- | --- | --- | --- | --- | --- | --- | --- |
| **Improved** |  |  |  |  |  |  |  |
| AGRIC | 12 | 83.53±4.20a-d | -0.69±0.54a-d | 29.63±2.80a-d | 4.49±0.84b-e | 83.36±4.18ab | 0.34±0.17a-f |
| IITA-TMS-IBA010040 | 12 | 79.28±8.56d | -1.69±1.12e | 25.95±4.83d | 4.91±1.04ab | 82.34±4.37ab | 0.14±0.12g |
| IITA-TMS-IBA30572 | 12 | 83.52±2.94a-d | -0.72±0.51a-d | 28.21±4.34b-d | 3.64±0.56fg | 78.64±6.60b | 0.19±0.06fg |
| IITA-TMS-IBA961632 | 12 | 86.15±2.47ab | -0.79±0.54b-e | 29.31±6.05a-d | 4.57±0.62a-d | 80.99±4.63ab | 0.40±0.13ab |
| IITA-TMS-IBA980505 | 12 | 85.07±1.79a-c | -0.80±0.38b-e | 27.52±3.34b-d | 4.38±0.59b-f | 80.83±4.53ab | 0.24±0.05b-g |
| IITA-TMS-IBA980581 | 12 | 82.21±5.08b-d | -0.36±0.70ab | 28.53±2.60b-d | 3.86±0.86d-g | 83.70±4.67a | 0.26±0.11a-g |
| NR8082 | 12 | 85.47±2.56a-c | -0.81±0.54b-e | 26.81±1.86cd | 4.47±0.69b-e | 84.41±4.20a | 0.31±0.06a-g |
| NWAGERI | 12 | 83.52±2.83a-d | -1.16±0.60b-e | 28.82±2.79a-d | 3.73±0.57e-g | 81.80±4.30ab | 0.22±0.12c-g |
| TMS13F1160P0004 | 12 | 86.88±2.89a | -0.68±0.74a-d | 29.48±4.76a-d | 4.25±0.71b-f | 80.94±4.63ab | 0.21±0.07d-g |
| TMS13F1176P0002 | 12 | 86.60±3.22a | -0.92±0.54b-e | 30.51±3.70a-c | 3.61±0.48fg | 83.82±4.04a | 0.37±0.10a-d |
| TMS13F1365P0002 | 12 | 83.73±3.40a-c | -1.13±0.94b-e | 30.58±2.83a-c | 4.08±0.60c-g | 82.76±4.22ab | 0.29±0.16a-g |
| WK195 | 12 | 81.69±3.91cd | -0.99±0.70b-e | 28.83±3.83a-d | 4.28±0.67b-f | 82.12±4.24ab | 0.37±0.14a-d |
| **Landraces** |  |  |  |  |  |  |  |
| CHIGAZU | 12 | 83.81±1.11a-c | -0.87±0.14b-e | 32.69±3.20a | 3.94±0.56d-g | 82.82±4.25ab | 0.40±0.03ab |
| DURUNGWO | 12 | 83.17±3.25a-d | -0.61±0.20a-d | 27.41±3.57b-d | 3.63±0.59fg | 83.31±4.32ab | 0.38±0.15a-c |
| KATIKATI | 12 | 82.72±2.95a-d | -0.50±0.84a-c | 29.69±1.73a-d | 3.91±0.55d-g | 82.86±4.19ab | 0.42±0.07a |
| MGBOTO_UMUAHIA | 12 | 84.75±2.29a-c | -1.54±1.18de | 29.73±1.62a-d | 3.44±0.56g | 84.33±4.20a | 0.19±0.11fg |
| NWOCHA | 12 | 85.54±3.41a-c | -0.64±0.59a-d | 30.93±1.87a-c | 4.06±0.61d-g | 80.32±4.91ab | 0.29±0.08a-g |
| SALOME(6MONTHS) | 12 | 85.51±3.72a-c | -0.71±0.94a-d | 28.28±2.30b-d | 3.86±0.73d-g | 83.53±4.46a | 0.23±0.11c-g |
| TMEB1 | 12 | 84.12±2.57a-c | 0.20±0.65a | 31.35±1.84ab | 4.34±0.57b-f | 82.86±4.43ab | 0.34±0.03a-f |
| TMEB2 | 12 | 83.48±3.88a-d | -1.46±0.59c-e | 28.80±4.44a-d | 4.92±1.15ab | 72.30±9.32c | 0.20±0.11 |
| TMEB419 | 12 | 83.19±3.16a-d | -0.22±0.48ab | 28.60±2.06a-d | 4.23±0.59b-f | 82.72±4.19ab | 0.40±0.09ab |
| TMEB693 | 12 | 83.25±3.56a-d | -1.34±1.18c-e | 29.31±6.57a-d | 4.85±0.83a-c | 72.32±9.48c | 0.22±0.16c-g |
| TMEB7 | 12 | 84.22±2.99a-c | -0.76±0.83b-e | 30.50±4.10a-c | 5.28±1.33a | 82.47±4.56ab | 0.27±0.12a-g |
| Improved varieties mean | | 83.97 | -0.89 | 28.68 | 4.19 | 82.14 | 0.28 |
| Landrace varieties mean | | 83.98 | -0.77 | 29.75 | 4.22 | 80.89 | 0.30 |
| Range |  | 67.84-91.72 | -4.02-1.16 | 18.35-38.98 | 2.72-7.58 | 59.56-89.26 | 0.05-0.65 |
| Year |  | *** | *** | *** | *** | *** | NS |
| Varieties |  | *** | *** | *** | *** | *** | *** |
| Year x Varieties |  | *** | *** | *** | NS | NS | NS |

**Table 6a**. Pearson correlation of the functional and chemical properties and the physical colour of gari compared with the root dry matter and gari yield from different varieties in Osun State

|  | Water absorption capacity | Dispersibility | Bulk density | Sugar content | Starch content | Total titratable acidity | Swelling power | Solubility index | L* | b* | a* | Root dry matter | Gari yield |
| --- | --- | --- | --- | --- | --- | --- | --- | --- | --- | --- | --- | --- | --- |
| Water absorption capacity | 1.00 |  |  |  |  |  |  |  |  |  |  |  |  |
| Dispersibility | -0.21 | 1.00 |  |  |  |  |  |  |  |  |  |  |  |
| Bulk density | 0.22 | -0.03 | 1.00 |  |  |  |  |  |  |  |  |  |  |
| Sugar content | -0.10 | -0.63** | 0.04 | 1.00 |  |  |  |  |  |  |  |  |  |
| Starch content | 0.39 | 0.20 | -0.29 | -0.39 | 1.00 |  |  |  |  |  |  |  |  |
| Total titratable acidity | -0.20 | 0.49* | -0.60** | -0.49* | 0.09 | 1.00 |  |  |  |  |  |  |  |
| Swelling power | -0.01 | -0.17 | 0.13 | 0.26 | -0.55* | 0.13 | 1.00 |  |  |  |  |  |  |
| Solubility index | 0.08 | -0.48* | 0.33 | 0.66** | -0.05 | -0.83** | -0.20 | 1.00 |  |  |  |  |  |
| L* | -0.25 | -0.27 | -0.03 | 0.23 | 0.08 | -0.36 | -0.35 | 0.25 | 1.00 |  |  |  |  |
| b* | -0.14 | -0.26 | 0.21 | 0.57** | -0.21 | -0.28 | 0.41 | 0.31 | -0.04 | 1.00 |  |  |  |
| a* | 0.29 | 0.60** | -0.16 | -0.76** | 0.42 | 0.52* | -0.03 | -0.63** | -0.41 | -0.43 | 1.00 |  |  |
| Root dry matter | -0.11 | -0.20 | 0.15 | 0.23 | 0.02 | -0.41 | -0.08 | 0.61** | -0.17 | 0.40 | -0.25 | 1.00 |  |
| Gari yield | -0.08 | 0.10 | 0.26 | -0.15 | 0.12 | -0.24 | -0.19 | 0.19 | 0.06 | 0.07 | 0.00 | 0.21 | 1.00 |

**Table 6b**. Pearson correlation of the functional and chemical properties and the physical colour of gari compared with the root dry matter and gari yield from different varieties in Imo State

| Parameters | Water absorption capacity | Dispersibility | Bulk density | Sugar content | Starch content | Total titratable acidity | Swelling power | Solubility index | L* | b* | a* | Root dry matter | Gari yield |
| --- | --- | --- | --- | --- | --- | --- | --- | --- | --- | --- | --- | --- | --- |
| Water absorption capacity | 1.00 |  |  |  |  |  |  |  |  |  |  |  |  |
| Dispersibility | -0.62** | 1.00 |  |  |  |  |  |  |  |  |  |  |  |
| Bulk density | -0.22 | 0.43* | 1.00 |  |  |  |  |  |  |  |  |  |  |
| Sugar content | 0.10 | 0.28 | -0.06 | 1.00 |  |  |  |  |  |  |  |  |  |
| Starch content | 0.02 | -0.11 | -0.02 | -0.58** | 1.00 |  |  |  |  |  |  |  |  |
| Total titratable acidity | 0.03 | 0.34 | -0.05 | 0.95** | -0.43* | 1.00 |  |  |  |  |  |  |  |
| Swelling power | 0.17 | -0.24 | -0.36 | -0.06 | 0.03 | -0.08 | 1.00 |  |  |  |  |  |  |
| Solubility index | -0.02 | -0.08 | -0.04 | -0.10 | 0.02 | -0.16 | -0.45* | 1.00 |  |  |  |  |  |
| L* | -0.58** | 0.36 | 0.39 | 0.03 | -0.06 | 0.08 | 0.10 | -0.24 | 1.00 |  |  |  |  |
| b* | -0.10 | 0.29 | -0.31 | 0.69** | -0.42* | 0.76** | 0.25 | -0.28 | 0.21 | 1.00 |  |  |  |
| a* | 0.01 | 0.29 | -0.06 | 0.83** | -0.36 | 0.91** | -0.18 | -0.24 | 0.12 | 0.68** | 1.00 |  |  |
| Root dry matter | -0.63** | 0.57** | 0.28 | -0.15 | 0.00 | -0.07 | -0.06 | -0.14 | 0.58** | 0.13 | -0.03 | 1.00 |  |
| Gari yield | -0.69** | 0.74** | 0.44* | -0.09 | 0.15 | 0.04 | -0.19 | -0.10 | 0.48* | 0.14 | 0.11 | 0.72** | 1.00 |
